# Micronutrients as Mitigators of Endocrine Disrupting Chemical Health Effects: A Scoping Review and Framework for Epidemiologic Studies

**DOI:** 10.1007/s40572-026-00536-8

**Published:** 2026-05-01

**Authors:** Heather M. Guetterman, Jessie P. Buckley, Kasandra Sanidad, Emily Jamo, Rita S. Strakovsky

**Affiliations:** 1 Department of Food Science and Human Nutrition, Michigan State University, 236C Trout Building, 469 Wilson Road, East Lansing, MI 517-353-3352 United States; 2 Institute for Integrative Toxicology, Michigan State University, East Lansing, MI United States; 3 Department of Epidemiology, Gillings School of Global Public Health, University of North Carolina at Chapel Hill, Chapel Hill, NC United States of America; 4 College of Osteopathic Medicine, Michigan State University, East Lansing, MI United States

**Keywords:** Endocrine disrupting chemicals, Micronutrients, Vitamins, Mitigation, Review

## Abstract

**Purpose of Review:**

Exposure to endocrine disrupting chemicals (EDC) is linked to numerous adverse health outcomes. However, limiting exposure to EDCs remains a significant challenge due to their widespread uses and persistence in the environment. Adequate micronutrient status supports optimal health and may offer actionable strategies for mitigating the adverse health effects of EDCs. This scoping review aimed to summarize the epidemiologic evidence on micronutrients as potential mitigators of EDC-related health outcomes, with the goal of guiding future research and methodologies.

**Recent Findings:**

We identified 71 epidemiologic studies assessing micronutrients as mitigators of EDC-outcome relations, focused primarily on exposures during pregnancy (*n* = 34). Most studies examined phthalates and/or environmental phenols (*n* = 25), per- and polyfluoroalkyl substances (*n* = 15), polycyclic aromatic hydrocarbons (*n* = 10), and self-reported pesticide exposure (*n* = 6). Most studies suggested higher levels of some micronutrients attenuated adverse associations of EDCs with some health outcomes, particularly iodine (thyroid hormones); folic acid (fertility, birth outcomes, neurodevelopment); vitamin D (lung function, neurodevelopment); and antioxidants (birth outcomes, aging, metabolic health). However, included studies assessed a wide range of micronutrients, EDCs, and outcomes, with limited overlap across studies.

**Summary:**

This scoping review identified few topics with substantial evidence to warrant focused systematic reviews, suggesting that additional prospective research is needed, especially in at-risk populations and sensitive periods outside of pregnancy. Future epidemiologic research should consider the co-occurrence of EDCs and micronutrients in foods and include multiple methods for assessing micronutrients. Finally, to strengthen causal inference, future research should thoughtfully model potential confounding, mediation, effect measure modification, and/or statistical interaction.

**Supplementary Information:**

The online version contains supplementary material available at 10.1007/s40572-026-00536-8.

## Introduction

Endocrine disrupting chemicals (EDCs) are environmental contaminants characterized by their ability to interfere with any aspect of hormone action [1]. Several hundred contaminants have endocrine disrupting properties (e.g., plasticizers, preservatives, flame retardants, pesticides), and their impact on human health varies across organ systems (e.g., metabolic disorders, reproductive health, cancer, thyroid disruption, neurodevelopmental outcomes) and depends on dose, duration, and timing of exposure during sensitive periods [2]. Mechanisms of action for many EDCs include interaction with nuclear receptors or steroid hormone (e.g., estrogen) receptor pathways, as well as mitochondrial dysfunction, oxidative stress, DNA methylation, histone modifications, and microRNA expression [2].

Exposure to EDCs can occur through dermal absorption, inhalation, and ingestion [2] – with food becoming contaminated during processing, from packaging, or through accumulation/absorption from the environment. Some chemicals (e.g., PFAS and persistent organic pollutants) are resistant to degradation and persist in the environment and human body long after exposure has ceased, with all individuals in NHANES having detectable blood levels [3, 4]. Other chemicals (e.g., phthalates and phenols) have short half-lives (< 48 h), and while they are rapidly excreted in urine, high detection frequencies of these non-persistent contaminants in individuals from the U.S. and other countries suggests constant exposure [5, 6]. Reducing exposure to EDCs is challenging given their ubiquitous presence in the environment and consumer products, difficulties in translating research into policy, and the replacement of chemicals with potentially more harmful alternatives [7, 8]. Therefore, identifying modifiable factors that mitigate the adverse impact of EDCs could represent strategies for reducing EDC-associated health risks. Additionally, these factors may help identify subpopulations who may be particularly susceptible to EDC exposure and may benefit most from targeted public health interventions. Identifying susceptible populations may also lead to better estimates of EDC-outcome associations, which often have small effect sizes.

### Micronutrients as Potential Mitigators of EDC Health Effects

High-quality, nutrient-rich diets are associated with reduced risk of many adverse health outcomes, including cardiovascular disease, type 2 diabetes, and cancer [9]. Together with macronutrients (carbohydrates, protein, and fat) and food-matrix effects (i.e., the chemical and structural components of whole foods and their molecular interactions) [10], micronutrients contribute to many of the health-promoting actions of high-quality diets [11, 12]. Micronutrients refer to vitamins (e.g., vitamin A, vitamin D, B-vitamins such as folate) and minerals (e.g., iron, calcium, iodine) and are essential at small amounts in the diet. Micronutrients support normal cellular function within an adequate range of intake, whereas both insufficient and excess intake can lead to adverse health outcomes [13–15]. Importantly, experimental animal studies suggest that adequate intake of essential vitamins and minerals may mitigate adverse health impacts of EDCs by counteracting/opposing EDC actions related to oxidative stress/antioxidant pathways [16, 17], endocrine pathways [18], or DNA methylation [19]. Additionally, EDCs may increase physiological requirements for micronutrients, suggesting micronutrient interventions could directly counteract EDC action on micronutrient status and subsequent health effects. For example, in epidemiologic studies, several pollutants have been associated with lower concentrations of vitamin D [20–23] and folate [23–26]. To that end, several prior reviews have explored the role of diet in the relation between EDCs and health outcomes. A recent scoping review examined antioxidant vitamins as mitigators of health effects of phthalate exposure in epidemiologic and animal studies [27]. A semi-structured review examined nutritional interventions – including micronutrient supplementation – in humans to mitigate the impacts of EDCs on reproductive and pregnancy outcomes [28]. Other reviews have been conducted with narrow scopes focused on biological mechanisms of specific EDCs, nutrients, and/or outcomes in humans and/or animals [29–33]. While these prior reviews have summarized potential mechanisms underpinning micronutrient-EDC interactions, the current review additionally focuses on methodologic considerations critical for modeling and interpreting findings from epidemiologic studies that aim to assess micronutrients as mitigators of EDC-health relations.

### Statistical Considerations for Epidemiologic Studies Evaluating Micronutrient Mitigation of EDC-Health Relations

In the context of this review, we define mitigation as the potential for micronutrients to influence the strength or direction, or account for a portion of associations between EDCs and health outcomes. For example, lower levels of micronutrients may exacerbate adverse associations, and higher levels may attenuate associations. In epidemiologic studies, this concept of mitigation can be examined as effect measure modification (EMM) or statistical interaction, and in some cases through mediation, if micronutrients lie on the causal pathway. These concepts are described in Table 1 and below. These are separate from additive or multiplicative effects, in which the combined effect of EDCs and micronutrients on an outcome is simply the sum or product of the independent effects, without interaction (e.g., the cumulative effect of EDC mixtures on an outcome [34]), which does not directly provide information about mitigation. EMM and statistical interaction are similar yet distinct concepts. EMM suggests the association between an EDC and outcome varies by levels of micronutrient status with one exposure being considered as primary. For example, mitigation is evident when the effect estimate for the association between an EDC and a health outcome is smaller in magnitude or null when micronutrient status is high compared to low. On the other hand, statistical interaction suggests joint (i.e., synergistic or antagonistic) associations between EDCs and micronutrients, with two exposures being considered [35, 36]. Interactive effects represent departures from additivity or multiplicativity, in which the combined effect is greater than (synergistic) or less than (antagonistic) the sum or product of the independent effects. For example, mitigation is evident if the effect estimate for associations of high levels of both the EDC and the micronutrient with a health outcome is smaller in magnitude or null compared to the effect estimate for being exposed to the EDC alone (i.e., antagonism). Importantly, the characterization of an effect as interactive depends on the scale of analysis (additive vs. multiplicative), which is discussed in more detail later. Statistical interaction does not necessary imply a biological interaction (i.e., underlying physical mechanism in which EDCs and micronutrients interact), which is not possible to directly determine within the context of an epidemiologic study [37]. Finally, mediation suggests the effect estimate for the association between an EDC and a health outcome is fully or partially explained by micronutrient status. For example, mediation is evident if the estimated indirect effect of an EDC on a health outcome operates through changes in micronutrient status. Mediation can occur with EMM or statistical interaction; if mediation is present in addition to EMM or statistical interaction but not accurately modeled, conditioning on micronutrient level (i.e., through adjustment, restriction, stratification) can result in biased effect estimates [38].

**Table 1:** Summary of effects relevant to mitigation

Effect	Description	Statistical approach	Best suited for public health interpretation
Effect measure modification	Effect of an EDC on outcome varies by levels of a micronutrient	Product terms or stratification with or without tests for heterogeneity^1^	Identify susceptible populations to EDC exposure that may benefit most from EDC-lowering interventions
Statistical interaction	Combined effect of an EDC and micronutrient on outcome is greater than (synergism) or less than (antagonism) the sum or product of the independent effects	Product terms or additive interaction metrics (e.g., relative excess risk due to interaction)^1^	Inform interventions targeting micronutrients and EDCs
Mediated effect	Effect of an EDC on outcome is fully or partially explained by micronutrient status, with or without interaction	Traditional or causal mediation	Inform micronutrient interventions to target mediators along EDC-health outcome pathways

^1^Presence of effect measure modification or statistical interaction is scale dependent (additive vs. multiplicative), i.e., presence of modification/interaction on one scale does not imply presence on the other scale

Statistical modeling approaches for interaction often use product terms in regression models (i.e., EDC x micronutrient). For EMM, studies may use product terms and/or stratification (i.e., EDC-outcome relation separately modeled by level of micronutrient) which is sometimes accompanied by a statistical test for heterogeneity (e.g., two-sample Z-test) [39, 40]. Stratification can provide unbiased estimates when confounder-outcome associations also vary by level of the modifier, and in such cases, may better capture EMM than a model with a single product term [39]. For example, the association of smoking with a health outcome may differ by level of micronutrient intake; stratification by micronutrient intake accommodates both EDC-outcome and smoking-outcome heterogeneity, whereas a product term only captures EDC-outcome heterogeneity leading to biased estimates [39]. Modeling approaches for mediation include traditional and causal mediation methods. Traditional methods use regression coefficients to decompose an exposure-outcome association into direct and indirect effects, whereas causal mediation methods, which are preferred over traditional methods, define natural direct and indirect effects within a counterfactual framework and can incorporate exposure-mediator interactions [41–43].

Interpreting EMM by micronutrient status only requires accounting for confounding of the EDC-outcome association, whereas interpreting causal statistical interaction (e.g., intervening on both EDC and micronutrient status) and causal mediation require additionally accounting for confounding of the micronutrient-outcome association [35, 36, 42]. Differences in the interpretation of results from EMM, statistical interaction, and mediation have important implications for public health. EMM is best suited for identifying susceptible populations to EDC exposure (e.g., those with low micronutrient status). Statistical interaction, on the other hand, can inform a joint high-risk profile and is best suited for informing potential interventions to target both micronutrients and EDCs. Finally, mediation is best suited to inform potential micronutrient interventions that intervene on an EDC-outcome pathway [35, 36]. Although these interpretations are not mutually exclusive, some mitigation approaches have distinct implications. For example, EMM focuses on confounder selection relevant to the EDC exposure and not the micronutrient modifier. Therefore, although lower levels of vitamin D, for example, may exacerbate the association between an EDC and health outcome, intervening on vitamin D may not attenuate the association if micronutrient status is confounded by other dietary or lifestyle factors. Rather, the presence of EMM in this example implies that EDC-reduction strategies may improve health to a greater extent in those with lower vitamin D status compared to those with higher vitamin D status. However, if the above example accounted for confounders of both vitamin D status and EDCs in a statistical interaction framework rather than EMM, the presence of a statistical interaction implies that intervening on both exposures would improve health to a greater extent than intervening on one alone. In the case of mediation, after accounting for confounders of both vitamin D status and EDCs, if lower vitamin D status explained a portion of the association between an EDC and health outcome, then intervening to increase vitamin D could mitigate adverse health outcomes by disrupting this mechanistic pathway (i.e., proportion eliminated).

Importantly, the presence or direction of EMM or statistical interaction depends on the scale being used when modeling binary or categorical outcomes (i.e., multiplicative scale [risk ratios] vs. additive scale [risk difference]) [35, 37]. If both exposures are associated with the outcome, then mathematically the absence of an interaction on one scale implies the presence of interaction on the other [37]. Although the multiplicative scale is most commonly reported in epidemiologic studies, the additive scale is considered more relevant for guiding public health recommendations, and is determined using metrics such as relative excess risk due to interaction [37]. If the baseline risk for an outcome differs between groups with high vs. low micronutrient intake, the risk ratio – calculated using baseline risk as the denominator – may distort the group being impacted most by EDC exposure. For example, individuals with low micronutrient status may have a higher baseline risk for developing a disease (15 cases per 100 people) compared to those with high micronutrient status (5 cases per 100 people). If higher EDC exposure increases the risk for a disease by 15% in the low-status group and 25% in the high-status group on the multiplicative scale, this would lead to 2.25 and 1.25 additional cases per 100 people on the additive scale, respectively. Results on the multiplicative scale suggest that EDCs have a greater relative impact in the high micronutrient status group, whereas results on the additive scale identified a greater absolute burden in the low micronutrient status group. From a public health perspective, prioritizing EDC-reduction efforts among individuals with low micronutrient status would avert the greatest number of disease cases.

In the context of the approaches described above for micronutrient mitigation of EDC-health relations, additional complexities arise given the common sources and overlapping absorption, excretion, and/or metabolic pathways of micronutrients and EDCs [23, 44–46]. First, micronutrients and EDCs may be positively or negatively correlated due to common dietary sources (e.g., vitamin D and PFAS in fish) [47, 48], differing dietary sources (e.g., micronutrient-poor processed foods contain high levels of phthalates) [47–49], or a disease or metabolic state altering the absorption or metabolism of both EDCs and micronutrients. In the case of high correlation (either positive or negative), including both micronutrient and EDC in a model for assessment of a product term increases the risk of multicollinearity and model non-convergence [50]. Second, both EDCs and micronutrients may be nonmonotonically associated with health outcomes; for example, BPA may be more harmful at low levels than higher levels [2], and tolerable upper limits of intake have been developed for several micronutrients [13]. Without carefully considering mitigation at multiple levels of the exposure and mitigator, product terms may lead to spurious results or may not capture the mitigating role of micronutrients [50]. These and other factors may require statistical approaches such as marginal structural models [51], mediation/interaction decomposition methods [52], or mixtures methods that account for co-occurring and collinear exposures [53–56].

Given the potential for micronutrient status to mitigate the relations between EDCs and human health outcomes – and the clear methodological challenges of addressing these questions in epidemiologic studies – we conducted a scoping review to summarize the breadth of epidemiologic evidence on the role of micronutrients as potential mitigators of EDC-related health effects. As part of the scoping review, we also summarized methodological approaches applied by prior studies. We then outlined research gaps and provided guidance on methodologies to inform and support future research and the development of public health policies.

## Methods

This scoping review was conducted using the framework proposed by Levac et al. (2010) [57] and followed the Preferred Reporting Items for Systematic Reviews and Meta-Analyses extension for scoping reviews (PRISMA-ScR) [58]. We did not register a protocol for this review. We identified studies using a search strategy in PubMed (Supplemental Table 1) and reviewing references from relevant articles. We screened articles in duplicate (HMG and KS/EJ) and extracted data using a standardized form in Covidence (covidence.org). Extracted data included methods for assessing EDCs, micronutrients, outcomes, and mitigation (i.e., EMM, interaction, mediation), as well as statistical approach (e.g., stratification, product terms). Epidemiologic studies in any population assessing EDCs, micronutrients, and health-related outcomes were included, provided the study assessed a micronutrient as part of EMM, statistical interaction, or mediation. Heavy metals and air pollutants were outside the scope of this review, given the extensive literature in this area [59–71]. Polycyclic aromatic hydrocarbons (PAH) are common air pollutants that can also be ingested in foods; we excluded studies examining PAH as air pollution (e.g., via personal air monitors, at e-waste sites) and included studies assessing PAH exposure from food or if PAH metabolites were measured in urine. Studies were summarized in narrative form and in tables.

## Overview of Included Studies

**Fig. 1:**
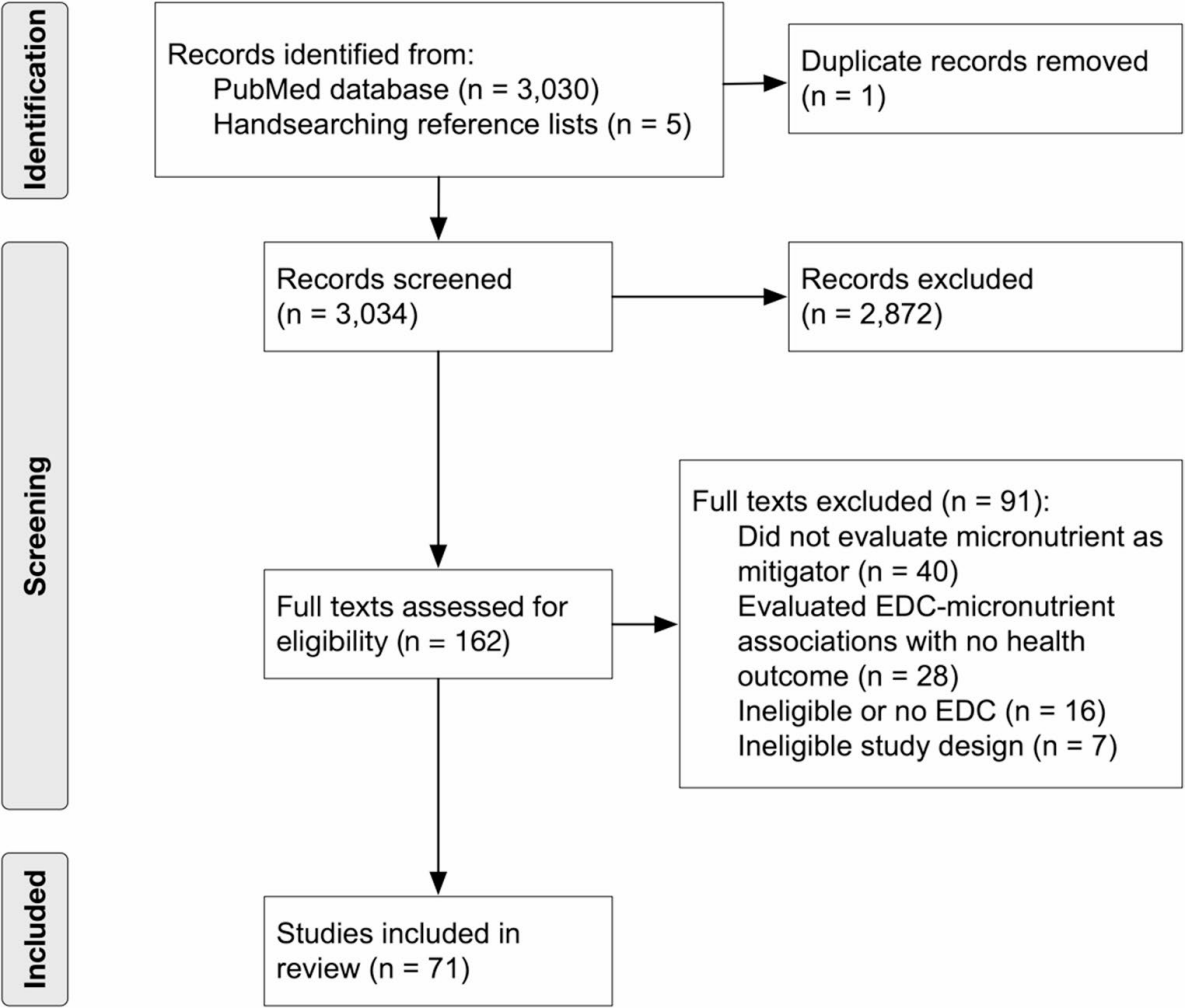
PRISMA flow chart

Results from screening are presented in Fig. 1. Of the 3,034 abstracts screened, we reviewed full texts for 162 and included 71 studies. An overview of results is provided in Table 2, and detailed methods and results of included studies is provided in Supplemental Table 2 and below.

**Table 2:**
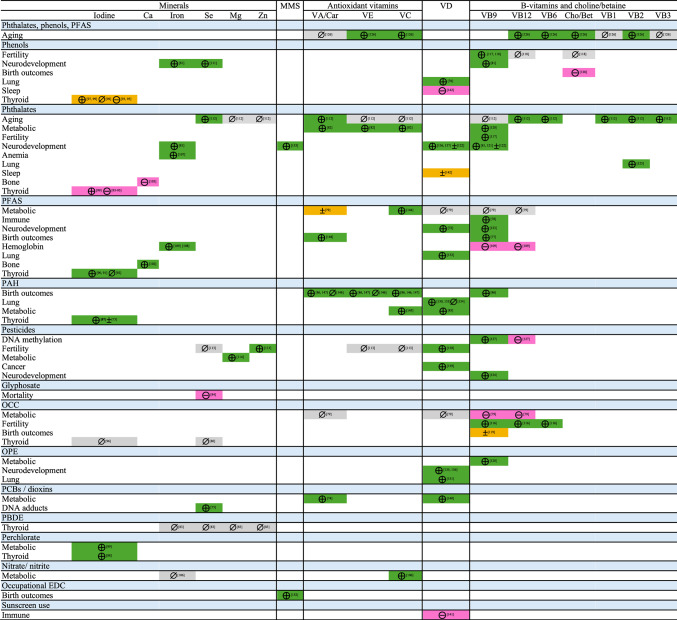
Overview of included studies

Summarized evidence across studies in each cell are shaded, green (attenuated in most studies), magenta (exacerbated in most studies), orange (mixed results across studies), gray (null results from most studies)

Evidence of micronutrient mitigation of associations between EDC and outcome from individual studies, ⊕ attenuated (green), ⊝ exacerbated (magenta), ± mixed (attenuated and exacerbated, orange), or ⊘ null (gray)

*Bet* betaine, *Ca* calcium, *Cho* choline, *EDC* endocrine-disrupting chemical, *OCC* organochlorine compound, *OPE* organophosphate ester, *PBDE* polybrominated diphenyl ether, *PCB* polychlorinated biphenyl, *PFAS* per- and polyfluoroalkyl substances, *Se* selenium, *VA/Car* vitamin A or carotenoids, *VB1* vitamin B1 (thiamin), *VB12* vitamin B12 (cobalamin), *VB2* vitamin B2 (riboflavin), *VB3* vitamin B3 (niacin), *VB6* vitamin B6, *VB9* vitamin B9 (folate/folic acid), *VC* vitamin C, *VE* vitamin E, *Zn* zinc

### Study Design, Setting, Population

Most studies were prospective (*n* = 34) or cross sectional (*n* = 33), followed by two studies with a case control design and two studies conducted within a randomized controlled trial. Most studies were conducted in the U.S. (*n* = 35) or China (*n* = 16), with others conducted in Canada (*n* = 5), Mexico (*n* = 2), countries in Europe (*n* = 9), or other countries in Asia (*n* = 4). Almost half of studies assessed exposures during pregnancy (*n* = 34 of 71), and almost all of cohort studies were conducted during pregnancy (*n* = 28 of 34). Studies included a wide range of ages, including children and/or adolescents (8–19 y; *n* = 7), adults (*n* = 29), both adults and children (*n* = 7), older adults (*n* = 1), and mother-child pairs (*n* = 27) with ages of children ranging from newborns to 9 years of age. Of these studies, some focused on specific populations, including one in children and adolescents with asthma, one in mother-twin pairs, two in Inuit populations, one in women receiving IVF treatments, 10 including outcomes in pregnant women, and four in populations with occupational-level exposure, including three among male pesticide applicators and one among female textile workers intending to become pregnant.

### Exposure Assessment: Endocrine-Disrupting Chemicals

Most studies focused on a single class of EDCs, measured in biological samples (urine, plasma/serum) or via questionnaire. The most commonly examined EDCs included PFAS (*n* = 13), PAH (*n* = 10; as benzo[a]pyrene (B(a)P)-DNA adducts, urinary metabolites, or B(a)P intake), phthalates (*n* = 10), environmental phenols (*n* = 6), both phthalates and phenols (*n* = 6), and self-reported exposure to household and/or occupational pesticides (*n* = 6). Four studies examined organochlorine pesticides (OCPs), including one study focusing on dichlorodiphenyltrichloroethane (DDT); three studies examined polychlorinated biphenyls (PCBs) and/or dioxins; two studies each examined organophosphate esters (OPEs) and perchlorate; and one study each examined polybrominated diphenyl ethers (PBDEs), glyphosate, nitrate/nitrite, self-reported occupational exposure to EDCs, and self-reported sunscreen use as a measure of benzophenone-3 exposure. Four studies assessed multiple classes of EDCs, including PFAS and OCPs (*n* = 1), phthalates, phenols, and PFAS (*n* = 1), and OPEs and phthalates (*n* = 2). A total of 17 studies assessed micronutrient mitigation using mixtures methods for EDCs (e.g., Bayesian kernel machine regression (BKMR), elastic net, quantile g-computation, weighted quantile sum), either stratifying the mixture by micronutrient level (*n* = 12) or including both micronutrients and EDCs within a mixture using BKMR or elastic net (*n* = 5).

### Mitigator Assessment: Micronutrients

Included studies examined micronutrients in serum/plasma/whole blood (*n* = 26), urine (*n* = 11), dietary intake with or without including supplements (*n* = 19), supplements only (*n* = 10), or both diet/supplements and serum/plasma/whole blood to assess multiple micronutrients (*n* = 1) or the same micronutrient (*n* = 2, plasma and dietary folate; *n* = 1, whole blood and dietary selenium). One study used genetic polymorphisms to estimate vitamin D status. Most studies focused on a single micronutrient, with vitamin D (*n* = 16), iodine (*n* = 14), and folate or folic acid (*n* = 8) as the most commonly assessed, followed by selenium (*n* = 3), calcium (*n* = 2), vitamin C (*n* = 2), iron (*n* = 2), and one study each for carotenoids, riboflavin (vitamin B2), magnesium, and betaine/choline. Five studies assessed two micronutrients (folate or folic acid plus iron, vitamin B12, or vitamin D; vitamin C plus iron; selenium plus copper) and 14 studies assessed three or more micronutrients, with two focusing on methyl donors (e.g., folate, B-vitamins, choline), one on essential metals, three on antioxidants (vitamins A, C, E, carotenoids), and seven assessing a broad range of micronutrients. Only two studies examined multiple-micronutrient supplementation.

### Summary of Outcomes

Studies examined a wide range of outcomes, with most focusing on thyroid function (*n* = 15) and neurocognitive, behavioral, and/or emotional development (*n* = 11), followed by lung function (*n* = 7), birth outcomes (*n* = 8), and fertility (*n* = 5). Several studies focused on metabolism-related outcomes (*n* = 11), including adiposity, glycemic control, cardiovascular disease, kidney function, liver function, and oxidative stress. Three studies focused on complete blood count (e.g., anemia/hemoglobin), two studies each focused on bone health, immune response, and aging, and one study each focused on cancer, mortality, sleep, DNA adducts, and DNA methylation.

### Statistical Approaches

Most studies in this review described analyses as EMM or conducted analyses consistent with EMM (e.g., stratification), whereas four conducted mediation analyses, and 9 studies assessed statistical interaction. Of the studies that assessed statistical interaction, only two described selection of confounders for micronutrient-outcome associations.

Fifty-four studies used product terms to assess mitigation, including three that examined statistical interaction as part of a machine learning approach (BKMR, adaptive elastic net selection), two that reported both multiplicative and additive interactions using the relative excess risk due to interaction (RERI), two that used BKMR to inform regression models with product terms and/or RERI, 32 that used stratification in addition to product terms, and one study, in which vitamin D and PFAS concentrations were strongly associated, that used matching methods to balance PFAS concentrations across vitamin D strata [72]. Thirteen studies used stratification only; of these, two reported results from statistical tests comparing stratified results (Cochran Q, two-sample Z-test). Four studies conducted causal mediation analyses but did not report whether exposure-mediator interactions were considered.

Several studies assessed potential nonlinearity (e.g., restricted cubic splines, quantiles, generalized additive models, Bayesian kernel machine regression) of EDC actions; however, few considered nonlinearity of micronutrient actions (*n* = 10) or cut-offs for excessive micronutrient intake (*n* = 1, i.e., excessive urinary iodine status) [73]. Of these studies, some used quadratic terms in models [74], nonlinear segmented regression [75], marginal effect plots [76], generalized additive models [77–80], and Johnson-Neyman plots [81], or used BKMR to assess nonlinearity of both EDCs and micronutrients [79, 80, 82, 83].

## Overview of Evidence for Micronutrient Mitigation of EDC Health Effects

Overall, most studies suggested that higher levels at least one micronutrient mitigated associations between EDCs and health outcomes, either based on formal statistical assessment (i.e., mediation analyses, product term, Cochran Q, two-sample Z-test; *n* = 57) or through qualitative assessment of strata-specific estimates in which the formal assessment was not significant (*n* = 7) or no formal statistical assessment was conducted (*n* = 8) (i.e., EDC associated with outcome in one strata but not the other). However, six of these studies and eight additional studies suggested that higher levels of at least one micronutrient exacerbated EDC-outcome associations. The remaining six studies did not identify a role of micronutrients in EDC-outcome associations in either direction. Of the 12 studies that conducted a formal statistical assessment and did not identify a significant statistical interaction or modification, some may have been underpowered (sample size as low as 164). For categorical outcomes, the absence of a multiplicative interaction implies the presence of an additive interaction if both the EDC and micronutrient are associated with the outcome [37], suggesting that some studies reporting null results using multiplicative interactions may have identified significant additive interactions [72, 84–86]. Few studies reported overlapping combinations of exposures, micronutrients, and outcomes, limiting the ability to compare across studies of specific EDC-micronutrient-outcome combinations. A summary of findings, by micronutrient, is provided in Table 2 and outlined below.

### Iodine

Iodine is critical for thyroid hormone synthesis [2]. Many EDCs have been shown to interfere with thyroid hormone metabolism and transport, and some (e.g., perchlorate) can inhibit iodine uptake in the thyroid [2]. A total of 13 studies examined iodine as a modifier of the association of EDCs with thyroid hormones (12 studies) and cardiovascular disease and associated risk factors (1 study), and one study examined iodine as a mediator of EDCs and thyroid volume. In children, lower urinary iodine mediated 8–15% of the associations between several PAH metabolites with higher thyroid volume [87]. Perchlorate was associated with thyroid hormones [88] and increased CVD risk [89] among adults and adolescents with lower iodine status but not in those with higher iodine status. Similarly, higher iodine intake attenuated the association between some PFAS and thyroid hormones in adolescents [90] and pregnant women [91] but not in adults [92]. However, phthalates [93, 94], phenols [93, 95], and PAH [73] were only associated with thyroid hormones at higher iodine levels in pregnant women, adults, and adolescents. In other studies during pregnancy, the association of OCPs [96] and BPA [97] with maternal thyroid hormones did not vary by iodine intake. Among studies measuring thyroid hormones in cord blood, the association of maternal phthalates and phenols with cord blood thyroid hormones did not vary by iodine status [97, 98], or were only associated in mothers with low iodine concentrations [91, 99], which may be sex-specific [97].

### Calcium

Calcium is required for bone development and maintenance, and circulating calcium is tightly regulated through parathyroid hormone and vitamin D-mediated processes [100]. Several EDCs may interfere with calcium homeostasis and bone health by disrupting estrogens, parathyroid hormone, or vitamin D metabolism [101]. Two studies assessed the role of calcium as a modifier of EDCs in relation to bone health. In one study, higher calcium intake attenuated the adverse association between PFAS and bone mineral content in adolescents [102], whereas in the second study, phthalates were associated with lower bone integrity in women who received calcium supplementation during pregnancy but not in the placebo group [103].

### Iron

Iron is required for hemoglobin synthesis, and iron deficiency has been associated with poor cognitive development and adverse birth outcomes [104]. Limited data suggest PFAS may interfere with hemoglobin structure [105]. Iron was examined as part of mediation in one study, statistical interaction in one study, and EMM in four studies, with heterogenous findings. In pesticide applicators and their spouses, heme iron intake, which can enhance nitrosation of nitrite, did not modify the association between nitrate/nitrite and end-stage renal disease [106]. Phthalates were associated with autistic traits in children whose mothers had low iron intake but not higher intake [81]. In adults, lower iron mediated 24–95% of the association between several phthalate metabolites and higher odds of anemia [107]. The associations of several PFAS with higher hemoglobin and hematocrit concentrations were attenuated in pregnant women who took iron supplements but not in non-supplement users [108], and in women with higher iron status measured via transferrin saturation but not with lower levels [109]. However, in the latter study, there was no evidence of statistical interaction when iron status was measured using serum ferritin [109].

### Other Minerals and Essential Metals

Several minerals, particularly selenium and zinc, have antioxidant properties [68], and others, such as magnesium, are involved in insulin and glucose metabolism and have been linked to lower incidence of type 2 diabetes [110], suggesting a role for minerals in mitigating EDC-related oxidative stress and metabolic outcomes. Of the eight studies assessing other minerals and essential metals, two assessed statistical interaction and six assessed EMM. Selenium was assessed in all but one of these studies, followed by zinc and magnesium in three studies, copper in two, and manganese, molybdenum, strontium, and cobalt each in one study. Selenium mitigated some adverse health outcomes related to phthalates and bisphenols, including maternal BPA and BPS with poorer motor function in children [111] and phthalate metabolites with aging acceleration [112]. Although mitigation was not formally assessed, selenium concentrations in whole blood were associated with lower DNA adducts in individuals with higher PCB concentrations [75]. Selenium did not modify associations between pesticide exposure and all-cause mortality [84] or infertility [113], whereas zinc [113] and magnesium [114] mitigated adverse associations of household pesticide use with higher odds of infertility and type 2 diabetes, respectively. Lastly, two studies focusing on thyroid hormones assessed nutrients and EDCs in BKMR analyses and did not identify evidence of statistical interactions by visual inspection of interaction plots, either among a mixture of plasma PBDEs and urinary essential and heavy metals [83] or among a mixture of cord blood selenium, OPEs, and mercury [80].

### B-Vitamins and Choline

As part of one-carbon metabolism, B-vitamins (vitamins B1 (thiamine), B2 (riboflavin), B3 (niacin), B6 (pyridoxine), B9 (folate), B12 (cobalamin)) and choline (and its derivatives, e.g., betaine) are critical for DNA synthesis and methylation [115], a potential mechanism of action for many EDCs [2, 19]. EDCs may compete for folate transporters or alter folate metabolism by depleting glutathione [23–26] and may alter endogenous choline synthesis by targeting estrogen pathways [2]. A total of 20 studies assessed B-vitamins and choline as mitigators of the relation between EDCs and health outcomes (folate/folic acid: 18; B12: 7; B6: 3; B1, B2, and/or B3: 2; betaine/choline: 3 studies), with four assessing statistical interaction, 15 assessing EMM, and one assessing mediation. Higher plasma B-vitamin status (vitamins B12, B6, folate), higher folate intake, or folic acid supplementation attenuated the association between DDT [116], bisphenols [117, 118], and/or phthalic acid [117] with adverse fertility-related outcomes (i.e., pregnancy loss, fecundability, implantation, clinical pregnancy). Maternal folic acid supplementation, higher folate intake, or higher plasma folate status attenuated associations of EDCs with lower birthweight and preterm birth (PFAS [78]; B(a)P DNA adducts [86]; PCB 118 [119]), adverse child and maternal liver enzymes (pesticides [120]), and risk of autistic traits and prosocial behavior difficulties (phthalates [81, 121, 122]; BPA [81]; PFOA, perfluorooctanoic acid [123]; pesticides [124]). In other studies, higher riboflavin intake attenuated the association of monobutyl phthalate with impaired lung function in adults [125], and higher red blood cell folate attenuated the association of PFAS with lower rubella and mumps antibodies in adolescents [78]. Intake of several B-vitamins (B2, B2, B3, B6, B12) attenuated the association between phthalate metabolites and aging acceleration [112], but not a mixture of phthalates, phenols, and PFAS on aging [126]. Although higher plasma folate attenuated associations between high pesticide exposure events and higher DNA methylation on GSTp1 (commonly hypermethylated in prostate cancer) in male pesticide applicators, higher plasma vitamin B12 concentrations strengthened this adverse association [127], and higher plasma folate and vitamin B12 concentrations strengthened associations of PFAS with higher hemoglobin concentrations [109] and persistent organic pollutants with higher offspring adiposity [79]. In mother-infant pairs, higher betaine and choline mediated the association between triclosan and lower head circumference [128].

### Vitamin D

Vitamin D has immunomodulatory and anti-inflammatory roles in the body, particularly related to respiratory diseases [129], and may mitigate EDC-induced oxidative stress and inflammation. Further, EDCs may disrupt vitamin D metabolism and circulating concentrations [20–23, 101]. A total of 18 studies assessed vitamin D as a mitigator of the associations of EDCs with a variety of health outcomes; three studies assessed statistical interaction and 15 assessed EMM. Higher vitamin D status attenuated the association between several EDCs, including BPA, OPE, PFAS, and PAH, with adverse lung function outcomes in children, adolescents, and/or adults [76, 130–133], including children with asthma [130]. However, prenatal vitamin D status did not modify the association between PAH exposure during pregnancy and asthma-related outcomes in children at age 6 years [134]. In several studies in the Ma’anshan birth cohort in China, maternal vitamin D sufficiency attenuated the association between PFAS, OPEs, and phthalates with poorer developmental and neurocognitive outcomes in children [72, 122, 135–137]. Higher vitamin D intake attenuated the association between household pesticide use and odds of infertility [138], and higher vitamin D status (estimated using variants in vitamin D-related genes) attenuated the association between pesticides and prostate cancer [139]. Although vitamin D status did not modify the relation of PAH with inflammation and albuminuria, a higher ratio of vitamin D to urinary PAH was associated with lower odds of inflammation and albuminuria [85]. Vitamin D sufficiency also mitigated the association between a mixture of dioxins and PCBs with kidney function measured as albumin-to-creatinine ratio, but not eGFR [140]. In contrast, there was no evidence of statistical interaction between vitamin D status and maternal persistent organic pollutants with childhood obesity [79], and vitamin D status may even exacerbate the association between frequent sunscreen use and odds of antinuclear antibodies [141] and a mixture of phthalates and phenols with lower sleep duration [142].

### Antioxidant Vitamins

Vitamins C, E, and A, as well as vitamin A-precursors and other carotenoids are commonly characterized as antioxidants due to their role in directly neutralizing free radicals (vitamin C, E) or indirectly regulating gene expression (vitamin A) [143]. In addition to inducing oxidative stress, EDCs can interact with vitamin A nuclear receptors, disrupting vitamin A signaling and gene expression [2, 23]. A total of 12 studies assessed the mitigating role of at least one vitamin commonly characterized as an antioxidant (vitamin C: 10, vitamin E: 7, vitamin A: 7, carotenoids: 6 studies), including three studies assessing statistical interaction, eight assessing EMM, and one assessing mediation. Studies suggest that antioxidants may mitigate the impact of PCBs, phthalates, phenols, and/or PFAS on biological aging [112, 126], insulin resistance/diabetes [74, 82, 144], and oxidative stress [144], including data from one randomized trial of vitamin C supplementation [144]. Several studies also suggest that antioxidants may mitigate the association between PAH exposure with maternal oxidative stress [145] and adverse birth outcomes [86, 146, 147], particularly lower birth weight [146, 147]. In mother-infant twin pairs, lower vitamin A mediated 36.9% of the association between intra-twin PFHpS exposure difference and higher birthweight difference [148]. Higher maternal concentrations of antioxidants either attenuated (beta-cryptoxanthin) or exacerbated (retinol, gamma-tocopherol) the association between maternal perfluorooctane sulfonate (PFOS) and higher risk of childhood overweight/obesity (interaction on the additive scale) [79]. The associations of household pesticide use with higher odds of infertility in women [113] and nitrate/nitrite with end-stage renal disease [106] did not vary by vitamin E and/or vitamin C intake.

### Multiple-Micronutrient Supplementation

Multiple-micronutrient supplements (i.e. multivitamin/multimineral supplements) are commonly taken by children and adults in the U.S [149]., and although evidence is limited for their beneficial effects in cardiovascular disease and cancer [150], they have been shown to reduce the risk of several adverse birth outcomes [151]. Two studies in mother-child pairs examined multiple-micronutrient supplementation as modifiers of EDC actions. In the first, prenatal multiple-micronutrient supplements attenuated the association of maternal occupational EDC exposure with higher odds of birth defects and congenital heart disease [152]. In the second study, maternal phthalate metabolites (MCPP, mono(3-carboxypropyl); MOP, mono-n-octyl; MCNP, mono-(carboxyisononyl)) were associated with higher odds of non-typical development in three-year-old children whose mothers did not take prenatal multiple-micronutrient supplements, whereas other metabolites (MCPP; MCOP, mono-carboxyisooctyl) were associated with lower odds of autism spectrum disorder in children whose mothers took prenatal multiple-micronutrient supplements [153].

## Summary and Recommendations to Guide Future Research Methodologies

Epidemiologic studies have assessed a wide range of micronutrients as mitigators of several EDC-health outcome relations, with minimal overlap to compare findings across studies. Several studies focused on iodine and thyroid hormones; folic acid and fertility, birth outcomes, or neurodevelopmental outcomes; vitamin D and lung function or neurodevelopmental outcomes; and antioxidants and birth outcomes or aging/metabolic health – highlighting potential topics for future systematic reviews. However, additional research is needed to generate evidence for synthesis across populations, micronutrients, EDCs, and outcomes to inform public health recommendations. Specifically, we identified the following understudied topics to inform research priorities. Most studies were conducted in adults or mother-child pairs, while other sensitive periods, such as puberty, postpartum, perimenopause, and older age were less represented. Studies are also needed in settings with a disproportionate burden of micronutrient deficiencies and EDC exposure, such as low- and middle-income countries and low-income, Black, and Latino communities [154–156]. For the greatest opportunity to inform public health, future research should focus on micronutrients with high prevalence of inadequate intake or insufficient status and with increased requirements or health risks during certain life stages, which will vary across populations (e.g., choline during pregnancy [157], vitamin B12 in older adults [158], calcium in adolescence [159] and peri/postmenopausal women [160]). Assessment of emerging contaminants of concern and chemical replacements will become increasingly important for future research [7, 161]. Lastly, although the most studied outcomes (i.e., thyroid function, neurocognitive development, and birth outcomes) still require further investigation, additional research is particularly warranted for several other common outcomes in which both EDCs and micronutrients are independent risk factors (e.g., male and female infertility, metabolic health, bone health [2, 101, 162–167].

Most studies applied EMM or statistical interaction without explicitly considering confounders of the micronutrient-outcome association. Therefore, for most studies, results suggest that those with lower micronutrient status/intake may be more susceptible to EDCs. Although most studies included several sociodemographic, dietary, and biological factors in analyses that may partially address confounding of micronutrient-outcome associations, the ability to interpret micronutrients as potential intervening factors – rather than solely as effect measure modifiers – is limited when confounders of the micronutrient-outcome association are not explicitly considered or accounted for in micronutrient assessment (i.e., genetic polymorphisms, randomized intervention). In such cases, observed associations could reflect underlying differences in health status, lifestyle, or other confounding factors rather than a true causal effect of micronutrients. Few studies examined micronutrients as mediators, and no studies assessed both mediation and statistical interaction/EMM. Some EDCs may disrupt metabolism and lower concentrations of iodine [2], vitamin D [20–23], and folate [23–26], suggesting these micronutrients may mediate EDC-health outcome associations. Therefore, results from studies assessing statistical interaction/EMM using biomarkers of these micronutrients may be biased by conditioning on a causal intermediate.

Many micronutrients co-occur in foods and with other healthy lifestyle factors. This may introduce confounding, difficulties teasing out the occurrence of additive or synergistic mitigation, or the possibility of mis-characterizing the true nutrient responsible for mitigation [168, 169]. No studies included in this review considered the co-occurrence or synergy of micronutrients in foods by examining mixtures of micronutrients, apart from those including both micronutrients and EDCs in a single mixtures model. However, although outside the scope of the current review, results from some included studies suggested that overall diet quality [102] and higher fruit/vegetables intake [145] – which typically track with micronutrients – may mitigate health effects of EDCs.

Although gold-standard methods exist for assessing biomarkers of persistent (e.g. blood) and non-persistent (e.g. urine) chemicals and many micronutrients, not all micronutrients have available biomarkers and measures of micronutrient status (biomarkers, dietary intake, supplements, genetic variants), including biomarkers, are susceptible to measurement error. For micronutrient assessment, using multiple methods with different sources of bias, such as both dietary intake and biomarkers, may help address measurement error in a single measure [170]. Only three studies assessed the same micronutrient using multiple methods (diet and biomarker), which confirmed the mitigating role of folate [78, 119] but not selenium [84]. In future systematic reviews, comparing results across studies by micronutrient assessment method would provide valuable information.

As we outlined in earlier sections and above, assessing micronutrient mitigation of EDC health effects comes with many analytical and methodological challenges. Based on these challenges and the methods of included studies (described above and in Supplemental Table 2), in Table 3, we provide a framework for assessing micronutrients as mitigators of EDC health effects in epidemiologic studies. Although not exhaustive, these considerations may inform future studies to address gaps identified above and contribute data for informing policies and interventions. Briefly, the first and most critical step is mapping out the potential relation between EDCs and micronutrients (e.g., overlapping exposure sources or metabolism) using tools such as directed acyclic graphs (DAG) to delineate a causal hypothesis. The research question and DAG will guide subsequent analysis steps, including the development of a data analysis plan to identify susceptible populations (EMM), inform interventions targeting micronutrients and EDCs (statistical interaction), and/or inform interventions to target mediators along EDC-health outcome pathways (mediation). To select appropriate modeling strategies, other considerations for the analysis plan include determining the presence of unmeasured confounders for micronutrients, confounder-outcome heterogeneity by micronutrient status, multicollinearity of micronutrients and EDCs, and reverse causality or time-varying factors. Although data availability/distribution will largely drive the operationalization of micronutrient data, researchers may consider triangulating evidence using multiple measurement methods (e.g., dietary intake, biomarkers) [170], mixtures of correlated micronutrients, and/or related exposures (e.g., food groups, diet patterns) [168, 171] – which can help address measurement error from a single measure and capture additive or synergistic mitigation. Researchers should carefully consider the full range of micronutrient values to assess potential nonlinearity and consider both clinically relevant and data-driven cut-points. Lastly, the reporting and interpretation of results should depend on the analysis (EMM, statistical interaction, and/or mediation) [35]. To improve integration of epidemiologic data into the U.S. Environmental Protection Agency’s (EPA) risk assessments, researchers should report subgroup-specific results to inform differential susceptibility, null results, results for non-transformed EDCs, and detailed distribution of EDCs, among other recommendations [8]. When translating results to dietary recommendations, researchers should consider co-occurrence of micronutrients and EDCs in the diet. For example, prior studies have highlighted that beneficial nutrients in fish may outweigh the risk of higher mercury exposure [172, 173].

**Table 3:** Summary of methodological considerations for epidemiologic analyses examining micronutrients as mitigators of endocrine disrupting chemicals on health outcomes

Analysis step	Considerations
Develop a directed acyclic graph (DAG)	• Represent effect measure modifiers and statistical interactions [174, 175] • Using prior literature, determine whether there are causal effects between EDCs and micronutrients that indicate mediation rather than or in addition to EMM or statistical interaction [176] • Include all common dietary sources of EDCs and micronutrients
Develop analysis plan	• Identify the mitigation approach based on the research goal: - Statistical interaction to inform interventions for both EDCs and micronutrients - EMM by micronutrient status to inform susceptible populations to EDCs or populations most likely to benefit from EDC interventions - EMM by EDC exposure to inform susceptible populations to micronutrients or populations most likely to benefit from micronutrient interventions - Mediation by micronutrient status to inform EDC-outcome mechanisms or to inform micronutrient interventions to target mediators of EDC-outcome pathways • Evaluate data availability for confounders of both micronutrients and EDCs; if missing critical confounders for micronutrients, consider EMM instead of statistical interaction • Conduct stratification analyses or include modifier-confounder interactions, in addition to modifier-exposure interactions, to avoid biased estimates when assessing EMM in which confounder-outcome associations vary by the modifier [39] • Consider causal mediation analysis using mediation/interaction decomposition methods to capture both mediation and potential statistical interaction, if applicable [41, 52] • Assess multicollinearity of micronutrients and EDCs: if micronutrients and EDCs are correlated, consider mixtures methods, if appropriate [53–56]; ensure analysis plan does not include scenarios that would extrapolate beyond the observed data (e.g., unable to assess low-high exposure scenario if exposures are strongly positively correlated) [53] • Consider marginal structural models to adjust for time-varying factors that may introduce reverse causality (e.g., micronutrient status and EDCs influence kidney function which also influences micronutrient status and EDCs) [51]; if longitudinal data on time-varying factors are not available, consider alternative measures of micronutrients and/or EDCs that are less affected by the outcome (e.g., biomarkers with longer half-lives, dietary intake)
Operationalize EDCs and micronutrients	• Examine other available measures of micronutrients to help address measurement error in a single measure (e.g., both dietary intake and biomarkers instead of biomarkers alone) [170] • Examine correlated micronutrients as a mixture to help better characterize key nutrients responsible for mitigation and identify additive or synergistic mitigation [53–56] • In addition to micronutrients, examine food groups, dietary patterns, or other lifestyle factors that track with micronutrient-rich diets, as these broader exposures may contain multiple components that act additively or synergistically to mitigate EDC health effects [168, 171] • Model across a full range of micronutrient values to assess potential nonlinear/nonmonotonic associations: - When assessing categorical micronutrient variables, evaluate both clinically-relevant cut-points (e.g., deficiency or insufficiency for biomarkers, inadequate and excessive micronutrient intake) and data-driven approaches (e.g., Johnson-Neyman, multivariable fractional polynomial interaction) to identify meaningful cut-points - When assessing continuous micronutrient variables, use flexible methods such as generalized additive models, splines, Johnson-Neyman, or multivariable fractional polynomial interaction to characterize nonlinearity
Report methods and results based on analytical approach	• For EMM: describe confounder selection for the primary exposure; present results stratified by the modifier, and on both the additive and multiplicative scales, if applicable [35] • For statistical interaction: describe confounder selection for both exposures; present results stratified by each exposure, and on both the additive and multiplicative scales, if applicable [35] • For mediation: describe confounder selection for exposure and mediator; specify the scale and whether exposure-mediator interactions were included; report natural direct and indirect effects as well as estimates for exposure-mediator and mediator-outcome relations; consider presenting proportion eliminated as a measure with more policy-relevance [176] • To improve integration of epidemiologic data into EPA’s risk assessments, report subgroup-specific results for differential susceptibility, null results, results for non-transformed EDCs, and detailed EDC distribution, among other recommendations [8]
Interpret results based on analytical approach to inform public health strategies	• For statistical interaction: if present, joint interventions targeting both EDCs and micronutrients yield greater benefits than a single intervention; if absent, interventions targeting one factor may still be effective without targeting both • For EMM by micronutrient status: if present, individuals with lower micronutrient status are more susceptible to health outcomes related to higher EDC exposure and may benefit most from EDC-reduction strategies; if absent, EDC-reduction strategies would be beneficial, regardless of micronutrient status • For EMM by EDC: if present, individuals with higher EDC exposure are more susceptible to health outcomes related to lower micronutrient status and may benefit most from micronutrient interventions; if absent, micronutrient interventions would be beneficial, regardless of EDC exposure levels • For mediation: if present, interventions targeting micronutrients may reduce the harmful impact of EDCs; if absent: interventions targeting EDCs may reduce their harmful impact directly or through other mechanisms • Consider co-occurrence of micronutrients and EDCs in the diet when translating results to dietary recommendations; for example, prior studies have highlighted that beneficial nutrients in fish may outweigh the risk of higher mercury exposure [172, 173]

*DAG* directed acyclic graph, *EDC* endocrine disrupting chemical, *EMM* effect measure modification, *EPA* U.S. Environmental Protection Agency

## Strengths and Limitations

This scoping review provided a comprehensive overview of micronutrients as potential mitigators of EDC-associated health outcomes in epidemiologic studies, with no limitations on population, micronutrient, or outcome – and identified several gaps in the literature to inform future research topics and methodological considerations. However, given the broad scope, we limited the search to a single database and excluded studies in animal models that may have identified micronutrient-EDC-outcome relations not yet assessed in humans that could have further informed future research and mechanisms. Studies are more likely to underreport statistically insignificant effect measure modification and statistical interaction [177] or may only report results as sensitivity analyses; therefore, although our search strategy was comprehensive, we may not have captured all relevant studies. We provided an overview of statistical approaches in included studies to guide interpretation of findings; however, our interpretation of results presented in this review may have varied across studies based on different p-value cut-offs, statistical methods, presentation of results (p-values for product terms and/or stratified analyses in text and/or tables) and number of associations assessed (type III error) by study authors. Further, we did not assess sources of bias in included studies or determine quality of evidence, as this was outside the scope of the review’s objectives which aimed to inform more focused systematic reviews. Future systematic reviews with narrower scopes are needed to synthesize evidence and understand sources of inconsistencies across studies (e.g. study design, sample size, population characteristics, exposure level, statistical methods).

## Conclusions

Overall, this scoping review identified 71 studies assessing micronutrients as modifiers or mitigators of EDC-outcome associations, with most suggesting that higher micronutrient status attenuates adverse associations of EDCs with numerous health outcomes across the lifespan. Included studies assessed a wide range of micronutrients, EDCs, and outcomes, with limited overlap across studies. Additional longitudinal data are needed in at-risk populations or sensitive periods outside of pregnancy, such as older age, perimenopause, and puberty. Future research should consider triangulating evidence using multiple methods for micronutrient assessment (diet, biomarkers, genetic variants). In addition to single nutrients, studies may also consider assessing dietary patterns or lifestyle factors that track with micronutrient-rich diets and mixtures of micronutrient intake/status – to understand which components of diets/lifestyles, individually or synergistically, are important mitigators. Finally, to strengthen causal inference and internal validity, future research should thoughtfully model potential confounding, mediation, effect measure modification, and statistical interaction. 

## Key References

- Knol MJ, VanderWeele TJ. Recommendations for presenting analyses of effect modification and interaction. Int J Epidemiol. 2012;41(2):514 − 20. doi: 10.1093/ije/dyr218.
  - ○ Guidance for reporting methods and results of effect measure modification and statistical interaction.
- VanderWeele TJ, Knol MJ. A Tutorial on Interaction. Epidemiologic Methods. 2014;3(1):33–72. doi: doi:10.1515/em-2013-0005.
  - ○ An overview of multiplicative versus additive interactions and application to various analyses.
- Shi B, Choirat C, Coull BA, VanderWeele TJ, Valeri L. CMAverse: A Suite of Functions for Reproducible Causal Mediation Analyses. Epidemiology. 2021;32(5):e20-e2. doi: 10.1097/ede.0000000000001378.
  - ○ A comprehensive R package for assessing mediation, including mediation/interaction decomposition, sensitivity analyses, and multiple mediators.
- Cano-Sancho G, Warembourg C, Güil N, Stratakis N, Lertxundi A, Irizar A, et al. Nutritional Modulation of Associations between Prenatal Exposure to Persistent Organic Pollutants and Childhood Obesity: A Prospective Cohort Study. Environ Health Perspect. 2023;131(3):37011. doi: 10.1289/EHP11258.
  - ○ An example of a study assessing micronutrient-EDC statistical interactions that explicitly stated confounder selection for micronutrients, included results on both the multiplicative and additive scale, considered nonlinearity using generalized additive models, and assessed mixtures of EDCs and micronutrients.

## Supplementary Information

Below is the link to the electronic supplementary material.

Supplementary Material 1 (XLXS 34.0 KB)

## Data Availability

No datasets were generated or analysed during the current study.

## References

[1] Zoeller RT, Brown TR, Doan LL, Gore AC, Skakkebaek NE, Soto AM, et al. Endocrine-Disrupting Chemicals and public health protection: a statement of principles from the endocrine society. Endocrinology. 2012;153(9):4097–110. 10.1210/en.2012-1422. 22733974 10.1210/en.2012-1422 PMC3423612

[2] Gore AC, Chappell VA, Fenton SE, Flaws JA, Nadal A, Prins GS, et al. EDC-2: The endocrine society’s second scientific statement on Endocrine-Disrupting Chemicals. Endocr Rev. 2015;36(6):E1–150. 10.1210/er.2015-1010. 26544531 10.1210/er.2015-1010 PMC4702494

[3] Xie X, Weng X, Liu S, Chen J, Guo X, Gao X, et al. Perfluoroalkyl and Polyfluoroalkyl substance exposure and association with sex hormone concentrations: results from the NHANES 2015–2016. Environ Sci Eur. 2021;33(1). 10.1186/s12302-021-00508-9. 10.1186/s12302-021-00508-9 PMC9440377 36061407

[4] Pumarega J, Gasull M, Lee DH, López T, Porta M. Number of persistent organic pollutants detected at high concentrations in blood samples of the United States population. PLoS ONE. 2016;11(8):e0160432. 10.1371/journal.pone.0160432. 27508420 10.1371/journal.pone.0160432 PMC4979965

[5] Calafat AM, Ye X, Wong LY, Bishop AM, Needham LL. Urinary concentrations of four parabens in the U.S. population: NHANES 2005–2006. Environ Health Perspect. 2010;118(5):679–85. 10.1289/ehp.0901560. 20056562 10.1289/ehp.0901560 PMC2866685

[6] Huang T, Saxena AR, Isganaitis E, James-Todd T. Gender and racial/ethnic differences in the associations of urinary phthalate metabolites with markers of diabetes risk: national health and nutrition examination survey 2001–2008. Environ Health. 2014;13(1):6. 10.1186/1476-069x-13-6. 24499162 10.1186/1476-069X-13-6 PMC3922428

[7] Hilz EN, Gore AC. Endocrine-Disrupting Chemicals: science and policy. Policy Insights Behav Brain Sci. 2023;10(2):142–50. 10.1177/23727322231196794. 39758979 10.1177/23727322231196794 PMC11698485

[8] Shaffer RM, Lee AL, Nachman R, Christensen K, Bateson TF. A perspective from US Environmental Protection Agency (EPA) scientists: how your epidemiologic analyses can inform the human health risk assessment process. Environ Health Perspect. 2025;133(3–4):045001. 10.1289/EHP15203. 40048177 10.1289/EHP15203 PMC12010935

[9] Schwingshackl L, Bogensberger B, Hoffmann G. Diet quality as assessed by the Healthy Eating Index, Alternate Healthy Eating Index, Dietary Approaches to Stop Hypertension Score, and Health Outcomes: an updated systematic review and meta-analysis of cohort studies. J Acad Nutr Diet. 2018;118(1):74–e10011. 10.1016/j.jand.2017.08.024. 29111090 10.1016/j.jand.2017.08.024

[10] Miller GD, Ragalie-Carr J, Torres-Gonzalez M, Perspective. Seeing the forest through the trees: the importance of food matrix in diet quality and human health. Adv Nutr. 2023;14(3):363–5. 10.1016/j.advnut.2023.03.005. 36934833 10.1016/j.advnut.2023.03.005 PMC10201811

[11] Aguilera-Méndez A, Boone-Villa D, Nieto-Aguilar R, Villafaña-Rauda S, Molina AS, Sobrevilla JV. Role of vitamins in the metabolic syndrome and cardiovascular disease. Pflugers Arch. 2022;474(1):117–40. 10.1007/s00424-021-02619-x. 34518916 10.1007/s00424-021-02619-x

[12] Fagbohun OF, Gillies CR, Murphy KPJ, Rupasinghe HPV. Role of antioxidant vitamins and other micronutrients on regulations of specific genes and signaling pathways in the prevention and treatment of cancer. Int J Mol Sci. 2023;24(7). 10.3390/ijms24076092. 10.3390/ijms24076092 PMC10093825 37047063

[13] Institute of Medicine (US). Subcommittee on interpretation and uses of dietary reference intakes, Institute of Medicine (US) standing committee on the scientific evaluation of Dietary Reference Intakes. DRI Dietary reference intakes: applications in dietary assessment. In: (US) NAP, editor. Washington (DC)2000. 25057725

[14] Pike V, Zlotkin S. Excess micronutrient intake: defining toxic effects and upper limits in vulnerable populations. Ann N Y Acad Sci. 2019;1446(1):21–43. 10.1111/nyas.13993. 30569544 10.1111/nyas.13993

[15] Verkaik-Kloosterman J, McCann MT, Hoekstra J, Verhagen H. Vitamins and minerals: issues associated with too low and too high population intakes. Food Nutr Res. 2012;56. 10.3402/fnr.v56i0.5728. 10.3402/fnr.v56i0.5728 PMC3321245 22489212

[16] Choi SM, Lim DS, Kim MK, Yoon S, Kacew S, Kim HS, et al. Inhibition of di(2-ethylhexyl) phthalate (DEHP)-induced endocrine disruption by co-treatment of vitamins C and E and their mechanism of action. J Toxicol Environ Health A. 2018;81(16):748–60. 10.1080/15287394.2018.1473262. 29842840 10.1080/15287394.2018.1473262

[17] Sahu C, Jena G. Combination treatment of zinc and selenium intervention ameliorated BPA-exposed germ cell damage in SD rats: elucidation of molecular mechanisms. Naunyn Schmiedebergs Arch Pharmacol. 2024;397(9):6685–704. 10.1007/s00210-024-03044-4. 38498059 10.1007/s00210-024-03044-4

[18] Wang Y, Chen B, Lin T, Wu S, Wei G. Protective effects of vitamin E against reproductive toxicity induced by di(2-ethylhexyl) phthalate via PPAR-dependent mechanisms. Toxicol Mech Methods. 2017;27(7):551–9. 10.1080/15376516.2017.1333556. 28532275 10.1080/15376516.2017.1333556

[19] Dolinoy DC, Huang D, Jirtle RL. Maternal nutrient supplementation counteracts bisphenol A-induced DNA hypomethylation in early development. Proc Natl Acad Sci U S A. 2007;104(32):13056–61. 10.1073/pnas.0703739104. 17670942 10.1073/pnas.0703739104 PMC1941790

[20] Mousavi SE, Amini H, Heydarpour P, Amini Chermahini F, Godderis L. Air pollution, environmental chemicals, and smoking may trigger vitamin D deficiency: evidence and potential mechanisms. Environ Int. 2019;122:67–90. 10.1016/j.envint.2018.11.052. 30509511 10.1016/j.envint.2018.11.052

[21] Chang C-J, Barr DB, Zhang Q, Dunlop AL, Smarr MM, Kannan K, et al. Associations of single and multiple per- and polyfluoroalkyl substance (PFAS) exposure with vitamin D biomarkers in African American women during pregnancy. Environ Res. 2021;202:111713. 10.1016/j.envres.2021.111713. 34284018 10.1016/j.envres.2021.111713 PMC8578284

[22] Etzel TM, Braun JM, Buckley JP. Associations of serum perfluoroalkyl substance and vitamin D biomarker concentrations in NHANES, 2003–2010. Int J Hyg Environ Health. 2019;222(2):262–9. 10.1016/j.ijheh.2018.11.003. 30503928 10.1016/j.ijheh.2018.11.003 PMC6408966

[23] Liu C, Zhou B, Huang L, Han D, He M, Zhou M, et al. Perfluoroalkyl and Polyfluoroalkyl Substances (PFAS) and vitamin metabolism: a nutritional perspective on an emerging environmental health issue. Nutrients. 2025;17(10). 10.3390/nu17101660. 10.3390/nu17101660 PMC12113770 40431401

[24] He X, Xue Q, Li D, Zhang S, Wu N, Li S, et al. Association between biomarkers of phthalate exposure and serum folate concentrations in children: a population-based cross-sectional study of the NHANES from 2011 to 2016. J Nutr. 2024;154(5):1596–603. 10.1016/j.tjnut.2024.03.008. 38484977 10.1016/j.tjnut.2024.03.008

[25] Jain RB. Impact of the increasing concentrations of selected perfluoroalkyl acids on the observed concentrations of red blood cell folate among US adults aged ≥ 20 years. Environ Sci Pollut Res Int. 2021;28(37):52357–69. 10.1007/s11356-021-14454-9. 34009570 10.1007/s11356-021-14454-9

[26] Tian Y, Luan M, Zhang J, Yang H, Wang Y, Chen H. Associations of single and multiple perfluoroalkyl substances exposure with folate among adolescents in NHANES 2007–2010. Chemosphere. 2022;307(Pt 3):135995. 10.1016/j.chemosphere.2022.135995. 35981617 10.1016/j.chemosphere.2022.135995

[27] Mo H-y, Shan C-h, Chen L-w, Chen X, Han C, Wu D, et al. Antioxidant vitamins’ modification of the adverse health effects induced by phthalate exposure: a scoping review of epidemiological and experimental studies. Ecotoxicol Environ Saf. 2024;286:117190. 10.1016/j.ecoenv.2024.117190. 39426110 10.1016/j.ecoenv.2024.117190

[28] Corbett GA, Lee S, Woodruff TJ, Hanson M, Hod M, Charlesworth AM, et al. Nutritional interventions to ameliorate the effect of endocrine disruptors on human reproductive health: a semi-structured review from FIGO. Int J Gynaecol Obstet. 2022;157(3):489–501. 10.1002/ijgo.14126. 35122246 10.1002/ijgo.14126 PMC9305939

[29] Grossklaus R, Liesenkötter K-P, Doubek K, Völzke H, Gaertner R. Iodine deficiency, maternal hypothyroxinemia and endocrine disrupters affecting fetal brain development: a scoping review. Nutrients. 2023. 10.3390/nu15102249. 37242131 10.3390/nu15102249 PMC10223865

[30] Nuttall JR. The plausibility of maternal toxicant exposure and nutritional status as contributing factors to the risk of autism spectrum disorders. Nutr Neurosci. 2017;20(4):209–18. 10.1080/1028415X.2015.1103437. 26613405 10.1080/1028415X.2015.1103437

[31] Wang Z, Zhou Y, Xiao X, Liu A, Wang S, Preston RJS, et al. Inflammation and cardiometabolic diseases induced by persistent organic pollutants and nutritional interventions: effects of multi-organ interactions. Environ Pollut. 2023;339:122756. 10.1016/j.envpol.2023.122756. 37844865 10.1016/j.envpol.2023.122756 PMC10842216

[32] Amjad S, Rahman MS, Pang MG. Role of antioxidants in Alleviating Bisphenol A Toxicity. Biomolecules. 2020;10(8). 10.3390/biom10081105. 10.3390/biom10081105 PMC7465987 32722388

[33] Bragg M, Chavarro JE, Hamra GB, Hart JE, Tabb LP, Weisskopf MG, et al. Prenatal diet as a modifier of environmental risk factors for autism and related neurodevelopmental outcomes. Curr Environ Health Rep. 2022;9(2):324–38. 10.1007/s40572-022-00347-7. 35305256 10.1007/s40572-022-00347-7 PMC9098668

[34] Carrico C, Gennings C, Wheeler DC, Factor-Litvak P. Characterization of weighted quantile sum regression for highly correlated data in a risk analysis setting. J Agric Biol Environ Stat. 2015;20(1):100–20. 10.1007/s13253-014-0180-3. 30505142 10.1007/s13253-014-0180-3 PMC6261506

[35] Knol MJ, VanderWeele TJ. Recommendations for presenting analyses of effect modification and interaction. Int J Epidemiol. 2012;41(2):514–20. 10.1093/ije/dyr218. 22253321 10.1093/ije/dyr218 PMC3324457

[36] VanderWeele TJ. On the distinction between interaction and effect modification. Epidemiology. 2009;20(6):863–71. 19806059 10.1097/EDE.0b013e3181ba333c

[37] VanderWeele TJ, Knol MJ. A Tutorial on interaction. Epidemiol Methods. 2014;3(1):33–72. 10.1515/em-2013-0005.

[38] Schisterman EF, Cole SR, Platt RW. Overadjustment bias and unnecessary adjustment in epidemiologic studies. Epidemiology. 2009;20(4):488–95. 10.1097/EDE.0b013e3181a819a1. 19525685 10.1097/EDE.0b013e3181a819a1 PMC2744485

[39] Buckley JP, Doherty BT, Keil AP, Engel SM. Statistical approaches for estimating sex-specific effects in endocrine disruptors research. Environ Health Perspect. 2017;125(6):067013. 10.1289/ehp334. 28665274 10.1289/EHP334 PMC5743445

[40] Paternoster RB, Mazerolle R, Piquero P. A. Using the correct statistical test for the equality of regression coefficients Criminology. 1998;36(4):859–66. 10.1111/j.1745-9125.1998.tb01268.x

[41] Shi B, Choirat C, Coull BA, VanderWeele TJ, Valeri L, CMAverse:. A suite of functions for reproducible causal mediation analyses. Epidemiology. 2021;32(5):e20–2. 10.1097/ede.0000000000001378. 34028370 10.1097/EDE.0000000000001378

[42] Rijnhart JJM, Lamp SJ, Valente MJ, MacKinnon DP, Twisk JWR, Heymans MW. Mediation analysis methods used in observational research: a scoping review and recommendations. BMC Med Res Methodol. 2021;21(1):226. 10.1186/s12874-021-01426-3. 34689754 10.1186/s12874-021-01426-3 PMC8543973

[43] Valeri L, Vanderweele TJ. Mediation analysis allowing for exposure-mediator interactions and causal interpretation: theoretical assumptions and implementation with SAS and SPSS macros. Psychol Methods. 2013;18(2):137–50. 10.1037/a0031034. 23379553 10.1037/a0031034 PMC3659198

[44] Kordas K, Lönnerdal B, Stoltzfus RJ. Interactions between nutrition and environmental exposures: effects on health outcomes in women and children1,2. J Nutr. 2007;137(12):2794–7. 10.1093/jn/137.12.2794. 18029501 10.1093/jn/137.12.2794

[45] Hoffman JB, Hennig B. Protective influence of healthful nutrition on mechanisms of environmental pollutant toxicity and disease risks. Ann N Y Acad Sci. 2017;1398(1):99–107. 10.1111/nyas.13365. 28574588 10.1111/nyas.13365 PMC5503778

[46] Cano-Sancho G, Casas M. Interactions between environmental pollutants and dietary nutrients: current evidence and implications in epidemiological research. J Epidemiol Commun Health. 2021;75(2):108. 10.1136/jech-2020-213789. 10.1136/jech-2020-213789 33023970

[47] Pacyga DC, Jolly L, Whalen J, Calafat AM, Braun JM, Schantz SL, et al. Exploring diet as a source of plasticizers in pregnancy and implications for maternal second-trimester metabolic health. Environ Res. 2024;263(Pt 3):120198. 10.1016/j.envres.2024.120198. 39427938 10.1016/j.envres.2024.120198 PMC11609028

[48] Paramasivam A, Murugan R, Jeraud M, Dakkumadugula A, Periyasamy R, Arjunan S. Additives in processed foods as a potential source of endocrine-disrupting chemicals: a review. J Xenobiot. 2024;14(4):1697–710. 10.3390/jox14040090. 39584955 10.3390/jox14040090 PMC11587131

[49] Buckley JP, Kim H, Wong E, Rebholz CM. Ultra-processed food consumption and exposure to phthalates and bisphenols in the US National Health and Nutrition Examination Survey, 2013–2014. Environ Int. 2019;131:105057. 10.1016/j.envint.2019.105057. 31398592 10.1016/j.envint.2019.105057 PMC6728187

[50] Duncan RP, Kefford BJ. Interactions in statistical models: three things to know. Methods Ecol Evol. 2021;12(12):2287–97. 10.1111/2041-210X.13714.

[51] Vanderweele TJ, Vansteelandt S, Robins JM. Marginal structural models for sufficient cause interactions. Am J Epidemiol. 2010;171(4):506–14. 10.1093/aje/kwp396. 20067916 10.1093/aje/kwp396 PMC2877448

[52] VanderWeele T. Explanation in causal inference: methods for mediation and interaction. Oxford, UNITED STATES: Oxford University Press, Incorporated; 2015.

[53] Joubert BR, Palmer G, Dunson D, Kioumourtzoglou MA, Coull BA. Workflow for statistical analysis of environmental mixtures. Environ Health Perspect. 2025. 10.1289/ehp16791. 42148041 10.1021/EHP.6c00155 PMC13151037

[54] Joubert BR, Kioumourtzoglou MA, Chamberlain T, Chen HY, Gennings C, Turyk ME, et al. Powering Research through Innovative Methods for Mixtures in Epidemiology (PRIME) program: novel and expanded statistical methods. Int J Environ Res Public Health. 2022;19(3). 10.3390/ijerph19031378. 10.3390/ijerph19031378 PMC8835015 35162394

[55] Hamra GB, Buckley JP. Environmental exposure mixtures: questions and methods to address them. Curr Epidemiol Rep. 2018;5(2):160–5. 10.1007/s40471-018-0145-0. 30643709 10.1007/s40471-018-0145-0 PMC6329601

[56] Gibson EA, Nunez Y, Abuawad A, Zota AR, Renzetti S, Devick KL, et al. An overview of methods to address distinct research questions on environmental mixtures: an application to persistent organic pollutants and leukocyte telomere length. Environ Health. 2019;18(1):76. 10.1186/s12940-019-0515-1. 31462251 10.1186/s12940-019-0515-1 PMC6714427

[57] Levac D, Colquhoun H, O’Brien KK. Scoping studies: advancing the methodology. Implement Sci. 2010;5:69. 10.1186/1748-5908-5-69. 20854677 10.1186/1748-5908-5-69 PMC2954944

[58] Tricco AC, Lillie E, Zarin W, O’Brien KK, Colquhoun H, Levac D, et al. PRISMA extension for scoping reviews (PRISMA-ScR): checklist and explanation. Ann Intern Med. 2018;169(7):467–73. 10.7326/M18-0850. 30178033 10.7326/M18-0850

[59] Péter S, Holguin F, Wood LG, Clougherty JE, Raederstorff D, Antal M, et al. Nutritional solutions to reduce risks of negative health impacts of air pollution. Nutrients. 2015;7(12):10398–416. 10.3390/nu7125539. 26690474 10.3390/nu7125539 PMC4690091

[60] Tong H. Dietary and pharmacological intervention to mitigate the cardiopulmonary effects of air pollution toxicity. Biochimica et Biophysica Acta (BBA) -. Gen Subj. 2016;1860(12):2891–8. 10.1016/j.bbagen.2016.05.014. 10.1016/j.bbagen.2016.05.014 27189803

[61] Singhal B, Chauhan S, Soni N, Gurjar V, Joshi V, Kaur P, et al. Modulatory effects of vitamin D: a possible approach to mitigate air pollution related pregnancy complications. J Reprod Infertil. 2024;25(2):79–101. 10.18502/jri.v25i2.16004. 39157803 10.18502/jri.v25i2.16004 PMC11327426

[62] Barthelemy J, Sanchez K, Miller MR, Khreis H. New opportunities to mitigate the burden of disease caused by traffic related air pollution: antioxidant-rich diets and supplements. Int J Environ Res Public Health. 2020. 10.3390/ijerph17020630. 31963738 10.3390/ijerph17020630 PMC7014349

[63] Ilaghi M, Kafi F, Shafiei M, Zangiabadian M, Nasiri MJ. Dietary supplementations to mitigate the cardiopulmonary effects of air pollution toxicity: a systematic review of clinical trials. PLoS ONE. 2024;19(6):e0304402. 10.1371/journal.pone.0304402. 38870164 10.1371/journal.pone.0304402 PMC11175466

[64] Whyand T, Hurst JR, Beckles M, Caplin ME. Pollution and respiratory disease: can diet or supplements help? A review. Respir Res. 2018;19(1):79. 10.1186/s12931-018-0785-0. 29716592 10.1186/s12931-018-0785-0 PMC5930792

[65] Miller CN, Rayalam S. The role of micronutrients in the response to ambient air pollutants: potential mechanisms and suggestions for research design. J Toxicol Environ Health Part B. 2017;20(1):38–53. 10.1080/10937404.2016.1261746. 10.1080/10937404.2016.1261746 PMC6130895 28145849

[66] Abuawad A, Bozack AK, Saxena R, Gamble MV. Nutrition, one-carbon metabolism and arsenic methylation. Toxicology. 2021;457:152803. 10.1016/j.tox.2021.152803. 33905762 10.1016/j.tox.2021.152803 PMC8349595

[67] Tinggi U, Perkins AV. Selenium status: its interactions with dietary mercury exposure and implications in human health. Nutrients. 2022. 10.3390/nu14245308. 36558469 10.3390/nu14245308 PMC9785339

[68] Rahman MM, Hossain KFB, Banik S, Sikder MT, Akter M, Bondad SEC, et al. Selenium and zinc protections against metal-(loids)-induced toxicity and disease manifestations: a review. Ecotoxicol Environ Saf. 2019;168:146–63. 10.1016/j.ecoenv.2018.10.054. 30384162 10.1016/j.ecoenv.2018.10.054

[69] Fisher M, Weiler HA, Kuiper JR, Borghese M, Buckley JP, Shutt R, et al. Vitamin D and toxic metals in pregnancy - a biological perspective. Curr Epidemiol Rep. 2024;11(3):153–63. 10.1007/s40471-024-00348-0. 39156920 10.1007/s40471-024-00348-0 PMC11329583

[70] Fan Y, Jiang X, Xiao Y, Li H, Chen J, Bai W. Natural antioxidants mitigate heavy metal induced reproductive toxicity: prospective mechanisms and biomarkers. Crit Rev Food Sci Nutr. 2024;64(31):11530–42. 10.1080/10408398.2023.2240399. 37526321 10.1080/10408398.2023.2240399

[71] Bae S, Kamynina E, Guetterman HM, Farinola AF, Caudill MA, Berry RJ, et al. Provision of folic acid for reducing arsenic toxicity in arsenic-exposed children and adults. Cochrane Database Syst Reviews. 2021;1010.1002/14651858.CD012649.pub2. 10.1002/14651858.CD012649.pub2 PMC8522704 34661903

[72] Gao Y, Zhang Y, Luo J, Mao D, Lei X, Liu C, et al. Effect modification by maternal vitamin D status in the association between prenatal exposure to per- and polyfluoroalkyl substances and neurodevelopment in 2-year-old children. Environ Int. 2024;185:108563. 10.1016/j.envint.2024.108563. 38461776 10.1016/j.envint.2024.108563

[73] Yang S, Sun J, Wang S, Zhang EL, Jiang S. Association of exposure to polycyclic aromatic hydrocarbons with thyroid hormones in adolescents and adults, and the influence of the iodine status. Environ Sci Process Impacts. 2023;25(9):1449–63. 10.1039/d3em00135k. 37555279 10.1039/d3em00135k

[74] Hofe CR, Feng L, Zephyr D, Stromberg AJ, Hennig B, Gaetke LM. Fruit and vegetable intake, as reflected by serum carotenoid concentrations, predicts reduced probability of polychlorinated biphenyl-associated risk for type 2 diabetes: National Health and Nutrition Examination Survey 2003–2004. Nutr Res. 2014;34(4):285–93. 10.1016/j.nutres.2014.02.001. 24774064 10.1016/j.nutres.2014.02.001 PMC4008967

[75] Ravoori S, Srinivasan C, Pereg D, Robertson LW, Ayotte P, Gupta RC. Protective effects of selenium against DNA adduct formation in Inuit environmentally exposed to PCBs. Environ Int. 2010;36(8):980–6. 10.1016/j.envint.2009.08.001. 19735942 10.1016/j.envint.2009.08.001 PMC3354714

[76] Sung M, Jee HM, Kim JH, Ha EK, Shin YH, Lim DH, et al. Serum vitamin D level mitigates fractional exhaled nitric oxide linked to bisphenol-A in school-aged children. Eur Rev Med Pharmacol Sci. 2022;26(5):1640–7. 10.26355/eurrev_202203_28232. 35302211 10.26355/eurrev_202203_28232

[77] Zhang Y, Mustieles V, Sun Q, Coull B, McElrath T, Rifas-Shiman SL, et al. Association of early pregnancy Perfluoroalkyl and Polyfluoroalkyl substance exposure with birth outcomes. JAMA Netw Open. 2023;6(5):e2314934–e. 10.1001/jamanetworkopen.2023.14934. 37256622 10.1001/jamanetworkopen.2023.14934 PMC10233420

[78] Zhang Y, Mustieles V, Wang YX, Sun Q, Coull B, Sun Y, et al. Red blood cell folate modifies the association between serum per- and Polyfluoroalkyl Substances and antibody concentrations in U.S. Adolescents. Environ Sci Technol. 2023;57(6):2445–56. 10.1021/acs.est.2c07152. 36715557 10.1021/acs.est.2c07152 PMC10539038

[79] Cano-Sancho G, Warembourg C, Güil N, Stratakis N, Lertxundi A, Irizar A, et al. Nutritional Modulation of Associations between prenatal exposure to persistent organic pollutants and childhood obesity: a prospective cohort study. Environ Health Perspect. 2023;131(3):37011. 10.1289/EHP11258. 36927187 10.1289/EHP11258 PMC10019508

[80] Wang J, Cao LL, Gao ZY, Zhang H, Liu JX, Wang SS, et al. Relationship between thyroid hormone parameters and exposure to a mixture of organochlorine pesticides, mercury and nutrients in the cord blood of newborns. Environ Pollut. 2022;292(Pt A):118362. 10.1016/j.envpol.2021.118362. 34648836 10.1016/j.envpol.2021.118362

[81] Uldbjerg CS, Leader J, Minguez-Alarcon L, Chagnon O, Dadd R, Ford J, et al. Associations of maternal and paternal preconception and maternal pregnancy urinary phthalate biomarker and bisphenol A concentrations with offspring autistic behaviors: The PEACE study. Environ Res. 2024;263(Pt 3):120253. 10.1016/j.envres.2024.120253. 39486680 10.1016/j.envres.2024.120253

[82] Li MC, Mínguez-Alarcón L, Bellavia A, Williams PL, James-Todd T, Hauser R, et al. Serum beta-carotene modifies the association between phthalate mixtures and insulin resistance: The National Health and Nutrition Examination Survey 2003–2006. Environ Res. 2019;178:108729. 10.1016/j.envres.2019.108729. 31521963 10.1016/j.envres.2019.108729 PMC6759414

[83] Hu MJ, Zhu JL, Zhang Q, He JL, Yang WJ, Zhu ZY, et al. Thyroid hormones in relation to polybrominated diphenyl ether and metals exposure among rural adult residents along the Yangtze River, China. Int J Hyg Environ Health. 2021;236:113800. 10.1016/j.ijheh.2021.113800. 34229161 10.1016/j.ijheh.2021.113800

[84] Chu PL, Hsiao CC, Su TC, Wang C, Lin CY. Urinary glyphosate, selenium status, and their impact on mortality: Evidence from NHANES 2013–2018. Ecotoxicol Environ Saf. 2025;292:117989. 10.1016/j.ecoenv.2025.117989. 40023997 10.1016/j.ecoenv.2025.117989

[85] Kadry AM, Lin YS, Caffrey JL, Sonawane B. Vitamin D status in relation to inflammatory risk and albuminuria associated with polycyclic aromatic hydrocarbon exposure in the US population. Arch Environ Occup Health. 2023;78(2):88–97. 10.1080/19338244.2022.2090890. 35766980 10.1080/19338244.2022.2090890 PMC11044198

[86] Zhao N, Wu W, Cui S, Li H, Feng Y, Guo L, et al. Effects of Benzo[a]pyrene-DNA adducts, dietary vitamins, folate, and carotene intakes on preterm birth: a nested case-control study from the birth cohort in China. Environ Health. 2022;21(1):48. 10.1186/s12940-022-00859-7. 35513839 10.1186/s12940-022-00859-7 PMC9074263

[87] Yang Z, Chen S, Zhou S, Xu C, Jing C, Guo C, et al. Association of polycyclic aromatic hydrocarbon internal exposure and urinary iodine concentration with thyroid volume in children. Environ Pollut. 2023;331(Pt 1):121912. 10.1016/j.envpol.2023.121912. 37247771 10.1016/j.envpol.2023.121912

[88] Blount BC, Pirkle JL, Osterloh JD, Valentin-Blasini L, Caldwell KL. Urinary perchlorate and thyroid hormone levels in adolescent and adult men and women living in the United States. Environ Health Perspect. 2006;114(12):1865–71. 10.1289/ehp.9466. 17185277 10.1289/ehp.9466 PMC1764147

[89] King L, Xia L, Chen J, Li W, Wang Q, Huang Y, et al. Exposure to perchlorate and cardiovascular disease in China: a community-based cross-sectional study and benchmark dose estimation. Environ Pollut. 2025;366:125429. 10.1016/j.envpol.2024.125429. 39617200 10.1016/j.envpol.2024.125429

[90] Freire C, Vela-Soria F, Castiello F, Salamanca-Fernández E, Quesada-Jiménez R, López-Alados MC, et al. Exposure to perfluoroalkyl substances (PFAS) and association with thyroid hormones in adolescent males. Int J Hyg Environ Health. 2023;252:114219. 10.1016/j.ijheh.2023.114219. 37451108 10.1016/j.ijheh.2023.114219

[91] Lebeaux RM, Doherty BT, Gallagher LG, Zoeller RT, Hoofnagle AN, Calafat AM, et al. Maternal serum perfluoroalkyl substance mixtures and thyroid hormone concentrations in maternal and cord sera: The HOME Study. Environ Res. 2020;185:109395. 10.1016/j.envres.2020.109395. 32222633 10.1016/j.envres.2020.109395 PMC7657649

[92] Webster GM, Rauch SA, Marie NS, Mattman A, Lanphear BP, Venners SA. Cross-sectional associations of serum perfluoroalkyl acids and thyroid hormones in U.S. adults: variation according to TPOAb and Iodine status (NHANES 2007–2008). Environ Health Perspect. 2016;124(7):935–42. 10.1289/ehp.1409589. 26517287 10.1289/ehp.1409589 PMC4937851

[93] Nakiwala D, Noyes PD, Faure P, Chovelon B, Corne C, Gauchez AS, et al. Phenol and phthalate effects on thyroid hormone levels during pregnancy: relying on in vitro assays and adverse outcome pathways to inform an epidemiological analysis. Environ Health Perspect. 2022;130(11):117004. 10.1289/EHP10239. 36350136 10.1289/EHP10239 PMC9645207

[94] Villanger GD, Drover SSM, Nethery RC, Thomsen C, Sakhi AK, Øvergaard KR, et al. Associations between urine phthalate metabolites and thyroid function in pregnant women and the influence of iodine status. Environ Int. 2020;137:105509. 10.1016/j.envint.2020.105509. 32044443 10.1016/j.envint.2020.105509

[95] Gao Q, Song Y, Jia Z, Huan C, Cao Q, Wang C, et al. Association of exposure to a mixture of phenols, parabens, and phthalates with altered serum thyroid hormone levels and the roles of iodine status and thyroid autoantibody status: a study among American adults. Ecotoxicol Environ Saf. 2024;282:116754. 10.1016/j.ecoenv.2024.116754. 39047362 10.1016/j.ecoenv.2024.116754

[96] Alvarez-Pedrerol M, Guxens M, Ibarluzea J, Rebagliato M, Rodriguez A, Espada M, et al. Organochlorine compounds, iodine intake, and thyroid hormone levels during pregnancy. Environ Sci Technol. 2009;43(20):7909–15. 10.1021/es9007273. 19921913 10.1021/es9007273

[97] Romano ME, Webster GM, Vuong AM, Thomas Zoeller R, Chen A, Hoofnagle AN, et al. Gestational urinary bisphenol A and maternal and newborn thyroid hormone concentrations: the HOME Study. Environ Res. 2015;138:453–60. 10.1016/j.envres.2015.03.003. 25794847 10.1016/j.envres.2015.03.003 PMC4403004

[98] Chevrier J, Gunier RB, Bradman A, Holland NT, Calafat AM, Eskenazi B, et al. Maternal urinary bisphenol a during pregnancy and maternal and neonatal thyroid function in the CHAMACOS study. Environ Health Perspect. 2013;121(1):138–44. 10.1289/ehp.1205092. 23052180 10.1289/ehp.1205092 PMC3553432

[99] Coiffier O, Nakiwala D, Rolland M, Malatesta A, Lyon-Caen S, Chovelon B, et al. Exposure to a mixture of non-persistent environmental chemicals and neonatal thyroid function in a cohort with improved exposure assessment. Environ Int. 2023;173:107840. 10.1016/j.envint.2023.107840. 36857904 10.1016/j.envint.2023.107840

[100] Veldurthy V, Wei R, Oz L, Dhawan P, Jeon YH, Christakos S. Vitamin D, calcium homeostasis and aging. Bone Res. 2016;4:16041. 10.1038/boneres.2016.41. 27790378 10.1038/boneres.2016.41 PMC5068478

[101] Park SY, Kong SH, Kim KJ, Ahn SH, Hong N, Ha J, et al. Effects of endocrine-disrupting chemicals on bone health. Endocrinol Metab (Seoul). 2024;39(4):539–51. 10.3803/EnM.2024.1963. 39015028 10.3803/EnM.2024.1963 PMC11375301

[102] Buckley JP, Zhou J, Marquess KM, Lanphear BP, Cecil KM, Chen A, et al. Per- and polyfluoroalkyl substances and bone mineral content in early adolescence: modification by diet and physical activity. Environ Res. 2024;252(Pt 1):118872. 10.1016/j.envres.2024.118872. 38580001 10.1016/j.envres.2024.118872 PMC11156547

[103] Cathey A, Tamayo-Ortiz M, Tamayo-Orozco J, Meeker JD, Peterson KE, Trejo-Valdivia B, et al. Calcium supplementation and body mass index modify associations between prenatal phthalate exposure and perinatal bone ultrasound measures among pregnant women. Environ Res. 2023;233:116513. 10.1016/j.envres.2023.116513. 37385416 10.1016/j.envres.2023.116513 PMC10529894

[104] Lynch S, Pfeiffer CM, Georgieff MK, Brittenham G, Fairweather-Tait S, Hurrell RF, et al. Biomarkers of Nutrition for Development (BOND)—Iron Review. J Nutr. 2018;148(suppl1):S1001–67. 10.1093/jn/nxx036. 10.1093/jn/nxx036 PMC6297556 29878148

[105] Perera NLD, Betancourt J, Miksovska J, O’Shea KE. Detail study on the interaction between perfluorooctanoic acid (PFOA) with human hemoglobin (Hb). Curr Res Toxicol. 2023;5:100130. 10.1016/j.crtox.2023.100130. 37822784 10.1016/j.crtox.2023.100130 PMC10563006

[106] Chen D, Parks CG, Beane Freeman LE, Hofmann JN, Sinha R, Madrigal JM, et al. Ingested nitrate and nitrite and end-stage renal disease in licensed pesticide applicators and spouses in the Agricultural Health Study. J Expo Sci Environ Epidemiol. 2024;34(2):322–32. 10.1038/s41370-023-00625-y. 38191926 10.1038/s41370-023-00625-y PMC11142909

[107] Ma H, Deng W, Liu J, Ding X. Association between urinary phthalate metabolites and Anemia in US adults. Sci Rep. 2024;14(1):21041. 10.1038/s41598-024-72147-y. 39251808 10.1038/s41598-024-72147-y PMC11385222

[108] Cui F, Liu H, Li Y, Zheng TZ, Xu S, Xia W, et al. Association of exposure to per- and polyfluoroalkyl substances with hemoglobin and hematocrit during pregnancy. Ecotoxicol Environ Saf. 2022;248:114319. 10.1016/j.ecoenv.2022.114319. 36423372 10.1016/j.ecoenv.2022.114319

[109] Lin CY, Lee HL, Wang C, Sung FC, Su TC. Examining the impact of polyfluoroalkyl substance exposure on erythrocyte profiles and its related nutrients: insights from a prospective study on young Taiwanese. Environ Pollut. 2024;359:124576. 10.1016/j.envpol.2024.124576. 39032552 10.1016/j.envpol.2024.124576

[110] Larsson SC, Wolk A. Magnesium intake and risk of type 2 diabetes: a meta-analysis. J Intern Med. 2007;262(2):208–14. 10.1111/j.1365-2796.2007.01840.x. 17645588 10.1111/j.1365-2796.2007.01840.x

[111] Liu J, Martin LJ, Dinu I, Field CJ, Dewey D, Martin JW. Interaction of prenatal bisphenols, maternal nutrients, and toxic metal exposures on neurodevelopment of 2-year-olds in the APrON cohort. Environ Int. 2021;155:106601. 10.1016/j.envint.2021.106601. 33962233 10.1016/j.envint.2021.106601

[112] Xu X, Zheng J, Li J, Shen Y, Zhu L, Jin Y, et al. Phthalate exposure and markers of biological aging: the mediating role of inflammation and moderating role of dietary nutrient intake. Ecotoxicol Environ Saf. 2024;281:116649. 10.1016/j.ecoenv.2024.116649. 38954910 10.1016/j.ecoenv.2024.116649

[113] Huang J, Hu L, Yang J. Dietary zinc intake and body mass index as modifiers of the association between household pesticide exposure and infertility among US women: a population-level study. Environ Sci Pollut Res Int. 2023;30(8):20327–36. 10.1007/s11356-022-23629-x. 36251185 10.1007/s11356-022-23629-x PMC9574790

[114] Huang J, Hu L, Yang J Dietary magnesium intake ameliorates the association between household pesticide exposure and type 2 diabetes: data from NHANES, 2007-2018. Front Nutr. 2022;9:903493. 10.3389/fnut.2022.903493. 35669066 10.3389/fnut.2022.903493 PMC9165529

[115] Stover PJ. Polymorphisms in 1-carbon metabolism, epigenetics and folate-related pathologies. J Nutrigenet Nutrigenomics. 2011;4(5):293–305. 10.1159/000334586. 22353665 10.1159/000334586 PMC3696357

[116] Ouyang F, Longnecker MP, Venners SA, Johnson S, Korrick S, Zhang J, et al. Preconception serum 1,1,1-trichloro-2,2,bis(p-chlorophenyl)ethane and B-vitamin status: independent and joint effects on women’s reproductive outcomes. Am J Clin Nutr. 2014;100(6):1470–8. 10.3945/ajcn.114.088377. 25411282 10.3945/ajcn.114.088377 PMC4232015

[117] Philips EM, Kahn LG, Jaddoe VWV, Shao Y, Asimakopoulos AG, Kannan K, et al. First trimester urinary bisphenol and phthalate concentrations and time to pregnancy: a population-based cohort analysis. J Clin Endocrinol Metab. 2018;103(9):3540–7. 10.1210/jc.2018-00855. 30016447 10.1210/jc.2018-00855 PMC6693040

[118] Mínguez-Alarcón L, Gaskins AJ, Chiu YH, Souter I, Williams PL, Calafat AM, et al. Dietary folate intake and modification of the association of urinary bisphenol A concentrations with in vitro fertilization outcomes among women from a fertility clinic. Reprod Toxicol. 2016;65:104–12. 10.1016/j.reprotox.2016.07.012. 27423903 10.1016/j.reprotox.2016.07.012 PMC5067190

[119] Hu JMY, Arbuckle TE, Janssen PA, Lanphear BP, Alampi JD, Braun JM, et al. Gestational exposure to organochlorine compounds and metals and infant birth weight: effect modification by maternal hardships. Environ Health. 2024;23(1):60. 10.1186/s12940-024-01095-x. 38951908 10.1186/s12940-024-01095-x PMC11218229

[120] India-Aldana S, Midya V, Betanzos-Robledo L, Yao M, Alcalá C, Andra SS, et al. Impact of metabolism-disrupting chemicals and folic acid supplementation on liver injury and steatosis in mother-child pairs. J Hepatol. 2024. 10.1016/j.jhep.2024.11.050. 39674324 10.1016/j.jhep.2024.11.050 PMC12086056

[121] Oulhote Y, Lanphear B, Braun JM, Webster GM, Arbuckle TE, Etzel T, et al. Gestational exposures to phthalates and folic acid, and autistic traits in canadian children. Environ Health Perspect. 2020;128(2):27004. 10.1289/EHP5621. 32073305 10.1289/EHP5621 PMC7064316

[122] Chen LW, Chen X, Han C, Zhu BB, Wang YF, Liu Y, et al. Modulation effects of folic acid and vitamin D on the relationships between prenatal cumulative phthalate exposure and preschoolers’ emotional and behavioral problems. Environ Int. 2025;196:109284. 10.1016/j.envint.2025.109284. 39889590 10.1016/j.envint.2025.109284

[123] Huang Y, Chen W, Gan Y, Liu X, Tian Y, Zhang J, et al. Prenatal exposure to per- and polyfluoroalkyl substances, genetic factors, and autistic traits: evidence from the Shanghai birth cohort. J Hazard Mater. 2024;480:135857. 10.1016/j.jhazmat.2024.135857. 39383700 10.1016/j.jhazmat.2024.135857

[124] Schmidt RJ, Kogan V, Shelton JF, Delwiche L, Hansen RL, Ozonoff S, et al. Combined prenatal pesticide exposure and folic acid intake in relation to autism spectrum disorder. Environ Health Perspect. 2017;125(9):097007. 10.1289/EHP604. 28934093 10.1289/EHP604 PMC5915192

[125] Lin J, Cheng S, Zhang J, Yuan S, Zhang L, Wu J, et al. The Association between daily dietary intake of riboflavin and lung function impairment related with dibutyl phthalate exposure and the possible mechanism. Nutrients. 2022;14(11). 10.3390/nu14112282. 10.3390/nu14112282 PMC9182752 35684081

[126] Huang W, Zhang Z, Colucci M, Deng L, Yang M, Huang X, et al. The mixed effect of Endocrine-Disrupting chemicals on biological age acceleration: unveiling the mechanism and potential intervention target. Environ Int. 2024;184:108447. 10.1016/j.envint.2024.108447. 38246039 10.1016/j.envint.2024.108447

[127] Rusiecki JA, Beane Freeman LE, Bonner MR, Alexander M, Chen L, Andreotti G, et al. High pesticide exposure events and DNA methylation among pesticide applicators in the agricultural health study. Environ Mol Mutagen. 2017;58(1):19–29. 10.1002/em.22067. 27996157 10.1002/em.22067 PMC5416937

[128] Zhang J, Wang Z, Dai Y, Zhang L, Guo J, Lv S, et al. Multiple mediation effects on association between prenatal triclosan exposure and birth outcomes. Environ Res. 2022;215:114226. 10.1016/j.envres.2022.114226. 36049513 10.1016/j.envres.2022.114226

[129] Gaudet M, Plesa M, Mogas A, Jalaleddine N, Hamid Q, Al Heialy S. Recent advances in vitamin D implications in chronic respiratory diseases. Respir Res. 2022;23(1):252. 10.1186/s12931-022-02147-x. 36117182 10.1186/s12931-022-02147-x PMC9483459

[130] Han YY, Rosser F, Forno E, Celedón JC. Exposure to polycyclic aromatic hydrocarbons, vitamin D, and lung function in children with asthma. Pediatr Pulmonol. 2018;53(10):1362–8. 10.1002/ppul.24084. 29943897 10.1002/ppul.24084 PMC6345525

[131] Aimuzi R, Dong C, Xie Z, Qu Y, Jiang Y, Luo K. Associations of urinary organophosphate esters metabolites with asthma and lung function in adolescents. J Expo Sci Environ Epidemiol. 2024;34(2):260–9. 10.1038/s41370-023-00540-2. 37019982 10.1038/s41370-023-00540-2

[132] Aker A, Courtemanche Y, Ayotte P, Robert P, Gaudreau É, Lemire M. Per and poly-fluoroalkyl substances and respiratory health in an Inuit community. Environ Health. 2024;23(1):83. 10.1186/s12940-024-01126-7. 39394583 10.1186/s12940-024-01126-7 PMC11470554

[133] Lin B, Liu W, Wang HH, Qian H, Zhu X, Xu M, et al. Associations of co-exposure to polycyclic aromatic hydrocarbons and vitamin D with early lung dysfunction: mediating roles of metabolic score-visceral adiposity index. Ecotoxicol Environ Saf. 2025;289:117496. 10.1016/j.ecoenv.2024.117496. 39657380 10.1016/j.ecoenv.2024.117496

[134] Loftus CT, Szpiro AA, Workman T, Wallace ER, Hazlehurst MF, Day DB, et al. Maternal exposure to urinary polycyclic aromatic hydrocarbons (PAH) in pregnancy and childhood asthma in a pooled multi-cohort study. Environ Int. 2022;170:107494. 10.1016/j.envint.2022.107494. 36279735 10.1016/j.envint.2022.107494 PMC9810359

[135] Li H, Tong J, Wang X, Lu M, Yang F, Gao H, et al. Associations of prenatal exposure to individual and mixed organophosphate esters with ADHD symptom trajectories in preschool children: the modifying effects of maternal Vitamin D. J Hazard Mater. 2024;478:135541. 10.1016/j.jhazmat.2024.135541. 39154480 10.1016/j.jhazmat.2024.135541

[136] Lu M, Gan H, Zhou Q, Han F, Wang X, Zhang F, et al. Trimester-specific effect of maternal co-exposure to organophosphate esters and phthalates on preschooler cognitive development: the moderating role of gestational vitamin D status. Environ Res. 2024;251(Pt 1):118536. 10.1016/j.envres.2024.118536. 38442813 10.1016/j.envres.2024.118536

[137] Gao H, Zhang C, Zhu B, Geng M, Tong J, Zhan Z, et al. Associating prenatal phthalate exposure with childhood autistic traits: investigating potential adverse outcome pathways and the modifying effects of maternal vitamin D. Eco Environ Health. 2024;3(4):425–35. 10.1016/j.eehl.2024.01.007. 39559191 10.1016/j.eehl.2024.01.007 PMC11570402

[138] Wu C, Xin X, Chen J. Vitamin D intake attenuated the association between pesticides exposure and female infertility. Clin Lab. 2023;69(9). 10.7754/Clin.Lab.2023.230201. 10.7754/Clin.Lab.2023.230201 37702693

[139] Karami S, Andreotti G, Koutros S, Barry KH, Moore LE, Han S, et al. Pesticide exposure and inherited variants in vitamin d pathway genes in relation to prostate cancer. Cancer Epidemiol Biomarkers Prev. 2013;22(9):1557–66. 10.1158/1055-9965.EPI-12-1454. 23833127 10.1158/1055-9965.EPI-12-1454 PMC3773544

[140] Zhang D, Qiao X, Peng J, Quan J, Huang Z, Yi B. Impact of dioxins and polychlorinated biphenyls on kidney parameters: the modulatory role of vitamin D. Ecotoxicol Environ Saf. 2025;294:118062. 10.1016/j.ecoenv.2025.118062. 40121944 10.1016/j.ecoenv.2025.118062

[141] Parks CG, Jusko TA, Meier HCS, Wilkerson J, Rider LG, Miller FW, et al. Sunscreen use associated with elevated prevalence of anti-nuclear antibodies in U.S. adults. J Autoimmun. 2024;149:103340. 10.1016/j.jaut.2024.103340. 39581147 10.1016/j.jaut.2024.103340 PMC11730459

[142] Zhou R, Chen Z, Yang T, Gu H, Yang X, Cheng S. Vitamin D deficiency exacerbates poor sleep outcomes with endocrine-disrupting chemicals exposure: a large american population study. Nutrients. 2024;16(9). 10.3390/nu16091291. 10.3390/nu16091291 PMC11085561 38732537

[143] Blaner WS, Shmarakov IO, Traber MG, Vitamin A, Vitamin E. Will the real antioxidant please stand up? Annu Rev Nutr. 2021;41:105–31. 10.1146/annurev-nutr-082018-124228. 34115520 10.1146/annurev-nutr-082018-124228

[144] Kim JH, Park HY, Jeon JD, Kho Y, Kim SK, Park MS, et al. The modifying effect of vitamin C on the association between perfluorinated compounds and insulin resistance in the Korean elderly: a double-blind, randomized, placebo-controlled crossover trial. Eur J Nutr. 2016;55(3):1011–20. 10.1007/s00394-015-0915-0. 25939797 10.1007/s00394-015-0915-0

[145] Kim H, Hwang JY, Ha EH, Park H, Ha M, Lee SH, et al. Fruit and vegetable intake influences the association between exposure to polycyclic aromatic hydrocarbons and a marker of oxidative stress in pregnant women. Eur J Clin Nutr. 2011;65(10):1118–25. 10.1038/ejcn.2011.77. 21587280 10.1038/ejcn.2011.77

[146] Duarte-Salles T, Mendez MA, Morales E, Bustamante M, Rodríguez-Vicente A, Kogevinas M, et al. Dietary benzo(a)pyrene and fetal growth: effect modification by vitamin C intake and glutathione S-transferase P1 polymorphism. Environ Int. 2012;45:1–8. 10.1016/j.envint.2012.04.002. 22565211 10.1016/j.envint.2012.04.002 PMC3855239

[147] Duarte-Salles T, Mendez MA, Meltzer HM, Alexander J, Haugen M. Dietary benzo(a)pyrene intake during pregnancy and birth weight: associations modified by vitamin C intakes in the Norwegian Mother and Child Cohort Study (MoBa). Environ Int. 2013;60:217–23. 10.1016/j.envint.2013.08.016. 24071023 10.1016/j.envint.2013.08.016

[148] Hu L, Huang Y, Shen X, Wang F, Yang M, Zhu M, et al. Cord serum metabolomic profiling associated with in utero exposure to per- and polyfluoroalkyl substances and birthweight discordance in twins: findings from Wuhan Twin Birth Cohort. Am J Epidemiol. 2025. 10.1093/aje/kwaf033. 40036346 10.1093/aje/kwaf033

[149] Cowan AE, Tooze JA, Gahche JJ, Eicher-Miller HA, Guenther PM, Dwyer JT, et al. Trends in overall and micronutrient-containing dietary supplement use in US adults and children, NHANES 2007–2018. J Nutr. 2023;152(12):2789–801. 10.1093/jn/nxac168. 35918260 10.1093/jn/nxac168 PMC9839985

[150] Fortmann SP, Burda BU, Senger CA, Lin JS, Whitlock EP. Vitamin and mineral supplements in the primary prevention of cardiovascular disease and cancer: an updated systematic evidence review for the U.S. preventive services task force. Ann Intern Med. 2013;159(12):824–34. 10.7326/0003-4819-159-12-201312170-00729. 24217421 10.7326/0003-4819-159-12-201312170-00729

[151] Keats EC, Haider BA, Tam E, Bhutta ZA. Multiple-micronutrient supplementation for women during pregnancy. Cochrane Database Syst Rev. 2019;3(3):Cd004905. 10.1002/14651858.CD004905.pub6. 30873598 10.1002/14651858.CD004905.pub6 PMC6418471

[152] Zhang H, Li Y, Zhang X, Chen W, Liang Q, Li C, et al. Potential occupational exposure of parents to endocrine disrupting chemicals, adverse birth outcomes, and the modification effects of multi-vitamins supplement and infant sex. Ecotoxicol Environ Saf. 2022;233:113314. 10.1016/j.ecoenv.2022.113314. 35189520 10.1016/j.ecoenv.2022.113314

[153] Shin HM, Schmidt RJ, Tancredi D, Barkoski J, Ozonoff S, Bennett DH, et al. Prenatal exposure to phthalates and autism spectrum disorder in the MARBLES study. Environ Health. 2018;17(1):85. 10.1186/s12940-018-0428-4. 30518373 10.1186/s12940-018-0428-4 PMC6280477

[154] Elegbeleye JA, Fayemi OE, Agbemavor WSK, Krishnamoorthy S, Adebowale OJ, Adeyanju AA, et al. Beyond calories: addressing micronutrient deficiencies in the world’s most vulnerable communities-a review. Nutrients. 2025;17(24). 10.3390/nu17243960. 10.3390/nu17243960 PMC12735482 41470905

[155] Ruiz D, Becerra M, Jagai JS, Ard K, Sargis RM. Disparities in environmental exposures to endocrine-disrupting chemicals and diabetes risk in vulnerable populations. Diabetes Care. 2018;41(1):193–205. 10.2337/dc16-2765. 29142003 10.2337/dc16-2765 PMC5741159

[156] Singh A, Singh G, Singh P, Mishra VK. Overview of sources, fate, and impact of endocrine disrupting compounds in environment and assessment of their regulatory policies across different continents. Total Environ Res Themes. 2023;7:100071. 10.1016/j.totert.2023.100071.

[157] Nguyen HT, Oktayani PPI, Lee SD, Huang LC. Choline in pregnant women: a systematic review and meta-analysis. Nutr Rev. 2025;83(2):e273–89. 10.1093/nutrit/nuae026. 38607338 10.1093/nutrit/nuae026

[158] Alvarez-Nuncio MDC, Ziegler TR. Micronutrient status and protein-energy malnutrition in free-living older adults: a current perspective. Curr Opin Gastroenterol. 2024;40(2):99–105. 10.1097/mog.0000000000001000. 38193299 10.1097/MOG.0000000000001000 PMC10872245

[159] Bailey ADL, Miketinas DC, London HE, Houslay T, Bender TM, Patterson AC Usual nutrient intake adequacy and nutritional status of United States children and adolescents: national health and nutrition examination survey 2001–March 2020. J Nutr. 2026;156(3):101377. 10.1016/j.tjnut.2026.101377. 41611129 10.1016/j.tjnut.2026.101377

[160] Marshall K, Teo L, Shanahan C, Legette L, Mitmesser SH. Inadequate calcium and vitamin D intake and osteoporosis risk in older Americans living in poverty with food insecurities. PLoS ONE. 2020;15(7):e0235042. 10.1371/journal.pone.0235042. 32639966 10.1371/journal.pone.0235042 PMC7343143

[161] Wang F, Xiang L, Sze-Yin Leung K, Elsner M, Zhang Y, Guo Y, et al. Emerging contaminants: a one health perspective. Innov (Camb). 2024;5(4):100612. 10.1016/j.xinn.2024.100612. 10.1016/j.xinn.2024.100612 PMC11096751 38756954

[162] Gaskins AJ, Chavarro JE. Diet and fertility: a review. Am J Obstet Gynecol. 2018;218(4):379–89. 10.1016/j.ajog.2017.08.010. 28844822 10.1016/j.ajog.2017.08.010 PMC5826784

[163] Kahn LG, Philippat C, Nakayama SF, Slama R, Trasande L. Endocrine-disrupting chemicals: implications for human health. Lancet Diabetes Endocrinol. 2020;8(8):703–18. 10.1016/S2213-8587(20)30129-7. 32707118 10.1016/S2213-8587(20)30129-7 PMC7437820

[164] Heindel JJ, Blumberg B, Cave M, Machtinger R, Mantovani A, Mendez MA, et al. Metabolism disrupting chemicals and metabolic disorders. Reprod Toxicol. 2017;68:3–33. 10.1016/j.reprotox.2016.10.001. 27760374 10.1016/j.reprotox.2016.10.001 PMC5365353

[165] Lanham-New SA. Importance of calcium, vitamin D and vitamin K for osteoporosis prevention and treatment: symposium on ‘Diet and bone health’. Proc Nutr Soc. 2008;67(2):163 – 76. 10.1017/S0029665108007003 18412990 10.1017/S0029665108007003

[166] Almoraie NM, Shatwan IM. The potential effects of dietary antioxidants in obesity: a comprehensive review of the literature. Healthc (Basel). 2024;12(4). 10.3390/healthcare12040416. 10.3390/healthcare12040416 PMC10887832 38391792

[167] Goncalves A, Amiot M-J. Fat-soluble micronutrients and metabolic syndrome. Curr Opin Clin Nutr Metabolic Care. 2017;20(6). 10.1097/MCO.0000000000000412 PMC5639995 28858890

[168] Jacobs DR Jr., Steffen LM. Nutrients, foods, and dietary patterns as exposures in research: a framework for food synergy. Am J Clin Nutr. 2003;78(3 Suppl):s508–13. 10.1093/ajcn/78.3.508S. 10.1093/ajcn/78.3.508S 12936941

[169] Bodnar LM, Cartus AR, Kirkpatrick SI, Himes KP, Kennedy EH, Simhan HN, et al. Machine learning as a strategy to account for dietary synergy: an illustration based on dietary intake and adverse pregnancy outcomes. Am J Clin Nutr. 2020;111(6):1235–43. 10.1093/ajcn/nqaa027. 32108865 10.1093/ajcn/nqaa027 PMC7266693

[170] Satija A, Yu E, Willett WC, Hu FB. Understanding nutritional epidemiology and its role in policy. Adv Nutr. 2015;6(1):5–18. 10.3945/an.114.007492. 25593140 10.3945/an.114.007492 PMC4288279

[171] Tapsell LC, Neale EP, Satija A, Hu FB. Foods, nutrients, and dietary patterns: interconnections and implications for dietary guidelines. Adv Nutr. 2016;7(3):445–54. 10.3945/an.115.011718. 27184272 10.3945/an.115.011718 PMC4863273

[172] Oken E, Radesky JS, Wright RO, Bellinger DC, Amarasiriwardena CJ, Kleinman KP, et al. Maternal fish intake during pregnancy, blood mercury levels, and child cognition at age 3 years in a US Cohort. Am J Epidemiol. 2008;167(10):1171–81. 10.1093/aje/kwn034. 18353804 10.1093/aje/kwn034 PMC2590872

[173] Oken E, Rifas-Shiman SL, Amarasiriwardena C, Jayawardene I, Bellinger DC, Hibbeln JR, et al. Maternal prenatal fish consumption and cognition in mid childhood: mercury, fatty acids, and selenium. Neurotoxicol Teratol. 2016;57:71–8. 10.1016/j.ntt.2016.07.001. 27381635 10.1016/j.ntt.2016.07.001 PMC5056822

[174] Attia J, Holliday E, Oldmeadow C. A proposal for capturing interaction and effect modification using DAGs. Int J Epidemiol. 2022;51(4):1047–53. 10.1093/ije/dyac126. 35696125 10.1093/ije/dyac126 PMC9365632

[175] Nilsson A, Bonander C, Strömberg U, Björk J. A directed acyclic graph for interactions. Int J Epidemiol. 2021;50(2):613–9. 10.1093/ije/dyaa211. 33221880 10.1093/ije/dyaa211 PMC8128466

[176] Lee H, Cashin AG, Lamb SE, Hopewell S, Vansteelandt S, VanderWeele TJ, et al. A guideline for reporting mediation analyses of randomized trials and observational studies: the AGReMA statement. JAMA. 2021;326(11):1045–56. 10.1001/jama.2021.14075. 34546296 10.1001/jama.2021.14075 PMC8974292

[177] Wang R, Lagakos SW, Ware JH, Hunter DJ, Drazen JM. Statistics in medicine — reporting of subgroup analyses in clinical trials. N Engl J Med. 2007;357(21):2189–94. 10.1056/NEJMsr077003. 18032770 10.1056/NEJMsr077003

